# Trends in Low-Value Health Service Use and Spending in the US Medicare Fee-for-Service Program, 2014-2018

**DOI:** 10.1001/jamanetworkopen.2020.37328

**Published:** 2021-02-16

**Authors:** John N. Mafi, Rachel O. Reid, Lesley H. Baseman, Scot Hickey, Mark Totten, Denis Agniel, A. Mark Fendrick, Catherine Sarkisian, Cheryl L. Damberg

**Affiliations:** 1 Division of General Internal Medicine and Health Services Research, Department of Medicine, David Geffen School of Medicine at the University of California, Los Angeles; 2 RAND Health Care, RAND Corporation, Santa Monica, California; 3 RAND Health Care, RAND Corporation, Boston, Massachusetts; 4 Division of General Internal Medicine and Primary Care, Brigham and Women’s Hospital, Boston, Massachusetts; 5 Harvard Medical School, Harvard University, Boston, Massachusetts; 6 Department of Internal Medicine, University of Michigan, Ann Arbor; 7 Center for Value-Based Insurance Design, University of Michigan, Ann Arbor; 8 Division of Geriatrics, David Geffen School of Medicine at the University of California, Los Angeles; 9 Geriatric Research Education and Clinical Center, VA Greater Los Angeles Healthcare System, Los Angeles, California

## Abstract

**Importance:**

Low-value care, defined as care offering no net benefit in specific clinical scenarios, is associated with harmful outcomes in patients and wasteful spending. Despite a national education campaign and increasing attention on reducing health care waste, recent trends in low-value care delivery remain unknown.

**Objective:**

To assess national trends in low-value care use and spending.

**Design, Setting, and Participants:**

In this cross-sectional study, analyses of low-value care use and spending from 2014 to 2018 were conducted using 100% Medicare fee-for-service enrollment and claims data. Included individuals were aged 65 years or older and continuously enrolled in Medicare parts A, B, and D during each measurement year and the previous year. Data were analyzed from September 2019 through December 2020.

**Exposure:**

Being enrolled in fee-for-service Medicare for a period of time, in years.

**Main Outcomes and Measures:**

The Milliman MedInsight Health Waste Calculator was used to assess 32 claims-based measures of low-value care associated with Choosing Wisely recommendations and other professional guidelines. The calculator designates services as wasteful, likely wasteful, or not wasteful based on an absence of indication of appropriate use in the claims history; calculator-designated wasteful services were defined as low-value care. Spending was calculated as claim-line level (ie, spending on the low-value service) and claim level (ie, spending on the low-value service plus associated services), adjusting for inflation.

**Results:**

Among 21 045 759 individuals with fee-for-service Medicare (mean [SD] age, 77.4 [7.9] years; 12 515 915 [59.5%] women), the percentage receiving any of 32 low-value services decreased from 36.3% (95% CI, 36.3%-36.4%) to 33.6% (95% CI, 33.6%-33.6%) from 2014 to 2018. Uses of low-value services per 1000 individuals decreased from 677.8 (95% CI, 676.2-679.5) to 632.7 (95% CI, 632.6-632.8) from 2014 to 2018. Three services comprised approximately two-thirds of uses among 32 low-value services per 1000 individuals: preoperative laboratory testing decreased from 213.8 (95% CI, 213.4-214.2) to 166.2 (95% CI, 166.2-166.2), while opioids for back pain increased from 154.4 (95% CI, 153.6-155.2) to 182.1 (95% CI, 182.1-182.1) and antibiotics for upper respiratory infections increased from 75.0 (95% CI, 75.0-75.1) to 82 (95% CI, 82.0-82.0). Spending per 1000 individuals on low-value care also decreased, from $52 765.5 (95% CI, $51 952.3-$53 578.6) to $46 921.7 (95% CI, $46 593.7-$47 249.7) at the claim-line level and from $160 070.4 (95% CI, $158 999.8-$161 141.0) to $144 741.1 (95% CI, $144 287.5-$145 194.7) at the claim level.

**Conclusions and Relevance:**

This cross-sectional study found that among individuals with fee-for-service Medicare receiving any of 32 measured services, low-value care use and spending decreased marginally from 2014 to 2018, despite a national education campaign in collaboration with clinician specialty societies and increased attention on low-value care. While most use of low-value care came from 3 services, 1 of these was opioid prescriptions, which increased over time despite the harms associated with their use. These findings may represent several opportunities to prevent patient harm and lower spending.

## Key Points

**Question:**

Have low-value care use and spending decreased over time with increasing focus on reducing waste in the US health care system?

**Findings:**

In this cross-sectional study of more than 21 million individuals with fee-for-service Medicare, the percentage receiving any of 32 measured low-value services decreased marginally from 2014 to 2018. Claim line–level spending on low-value care per 1000 individuals did not decrease substantially over this period.

**Meaning:**

This study found that among individuals with fee-for-service Medicare receiving any of 32 measured services, low-value care use and spending decreased marginally from 2014 to 2018, despite a national education campaign to address low-value care and increased attention on reducing health care waste.

## Teaser

This cross-sectional study examines changes in low-value care use and spending from 2014 to 2018 among the fee-for-service Medicare population.

## Introduction

An estimated 10% to 20% of health care spending consists of low-value care, defined as patient care that offers no net benefit in specific clinical scenarios (eg, antibiotic prescriptions for uncomplicated acute upper respiratory infections).^1,2,3,4^ Low-value care merits attention because it is associated with harmful outcomes in patients; for example, approximately 1 in every 1000 antibiotic prescriptions is associated with a serious complication for the patient (eg, infectious colitis) requiring an emergency department visit.^5^ Low-value care is also associated with wasteful spending and increased cost of care for patients and purchasers.^6,7^ Nevertheless, despite increasing awareness of low-value care and its associated harms, such care remains a common fixture in US medicine.^6,8,9,10,11,12,13,14,15^

To address this problem, in 2012 the American Board of Internal Medicine Foundation joined multiple clinician specialty societies to lead Choosing Wisely, an educational campaign to raise clinician and patient awareness about the problem of low-value care.^2^ The campaign received widespread attention among physicians, consumer groups, and policy makers, including the Medicare Payment Advisory Commission (MedPAC), which began monitoring low-value care use and spending in 2015.^16^ Additionally, public and private payers have increasingly adopted advanced payment models that hold clinicians accountable for total cost of care in an effort to reduce wasteful spending.^17,18,19,20,21,22^

Studies examining early trends^23,24,25,26,27^ found that low-value care use has remained similar or declined slightly over time since the Choosing Wisely campaign began; however, these studies included only data as recent as those from 2015 and did not focus on low-value care use among individuals with fee-for-service Medicare. The Medicare fee-for-service population is uniquely at risk for receiving low-value care owing to a combination of these individuals’ advanced age and multimorbidity, their generallys high use of health services, and the presence of the fee-for-service payment model.^6^ Examining recent national trends on low-value care use and spending in the Medicare program could help inform policy interventions that may safely lower spending, minimize patient harm, and improve quality of care. Accordingly, we sought to examine trends in low-value care use and associated spending among individuals with fee-for-service Medicare from 2014 to 2018.

## Methods

The RAND Institutional Review Board approved this cross-sectional study and determined that it was exempt from human participant research review and that informed consent could be waived because this was a secondary data analysis in which data were deidentified. Our study conforms to the Strengthening the Reporting of Observational Studies in Epidemiology (STROBE) reporting guideline.

### Data Sources and Participants

We performed a descriptive trends analysis assessing low-value service use and spending among individuals with 100% fee-for-service Medicare aged 65 years or older from 2014 to 2018. Specifically, this study analyzed 5 repeated annual cross-sectional groups, staggered over time from 2014 to 2018, although 8 601 535 of 21 045 759 individuals (40.9%) were included longitudinally across the 5-year study period (eFigure in the Supplement).^28^ We obtained demographic information, including race and ethnicity data, from the enrollment files. We used the carrier, outpatient, and patient claims files, as well as Medicare Part D event files, to quantify beneficiary-level low-value care use and associated spending. To create a stable and homogeneous study population for comparison across years, we used enrollment files to include individuals who were continuously enrolled in Medicare Parts A, B, and D for 2 years in each study year (ie, the measurement year and the year prior). To further ensure consistent comparisons across years, we included no more than 3 years of look-back of claims history preceding each measurement year (eg, the 2014 measurement year looks back for clinical indications as far as 2011, and the 2015 measurement year looks back as far as 2012).

### Measures of Low-Value Care

We examined claims-based low-value care measures from the Milliman MedInsight Health Waste Calculator version 7.1, a proprietary, algorithm-based software program that designates care as wasteful, likely wasteful, or not wasteful, reflecting recommendations from the Choosing Wisely campaign and other professional physician society guidelines.^7,29,30^ For example, antibiotic prescriptions for uncomplicated upper respiratory infections would require the absence of 4705 *International Classification of Diseases, Ninth Revision, Clinical modification* (*ICD-9-CM*)^31^ or *International Statistical Classification of Diseases, Tenth Revision, Clinical Modification* (*ICD-10-CM*)^32^ diagnosis codes (eg, diagnosis codes for chronic obstructive pulmonary disease or HIV) to be labeled as wasteful (eTable 1 in the Supplement). The software algorithm is updated annually with new clinician professional society guidelines; *ICD-10-CM*^32^ diagnosis codes; American Medical Association procedural codes; and National Drug Code entries. Results from the calculator have been used across multiple US states and purchasers and published in peer-reviewed journals.^7,29,30,33,34,35^ We limited our analysis to 32 measures (of 48 total measures) that were applicable to the older Medicare population (eg, excluding pediatric-oriented measures) and that lacked major *Current Procedural Terminology* (*CPT*) coding changes that precluded reliable trends analysis. (For example, in 2015, several *CPT* codes for vertebroplasty were removed from use, precluding a reliable trends analysis for this measure.)

To reduce risks associated with misclassification error, specifically the false labeling of care as low value when it was appropriate, we conservatively defined low-value care by including only the wasteful category; we did not label likely wasteful services as low-value care. Additionally, we limited our analysis to services for which there was sufficient claims history available for the individual so that we could determine the presence or absence of applicable clinical exclusions for designating a service as wasteful, likely wasteful, or not wasteful. For example, to determine whether imaging for headache was low value for an individual, we required at least 12 months of historical claims data for that individual to ensure that no cancer or head trauma diagnosis had been made in the previous year that would make use of imaging not wasteful (eTable 1 in the Supplement). We also excluded low-value care use and spending billed in the inpatient file from this analysis because we could not accurately associate spending with the low-value service itself.

### Spending Calculations

To quantify spending associated with each low-value service, we calculated allowed amounts, defined as the amount clinicians were paid for services, including patients’ out-of-pocket costs, in accordance with Research Data Assistance Center guidelines by applicable claim type.^36^ For the outpatient file, revenue center–level spending was used in lieu of claim line–level spending.

The Milliman MedInsight Health Waste Calculator uses 2 methods to quantify spending associated with low-value care, and we included the 2 spending estimation methods to illustrate a range of spending estimates.^35^ First, we used claim line–level spending, which is defined as spending associated with the low-value service itself as identified on the claim line; second, we used claim-level spending, which is broader and includes spending associated with the low-value service and associated services. More specifically, the claim-level spending method includes spending from all claim identification numbers in which at least 1 line has a service identified as associated with a low-value service. The claim line–level spending method counts only spending from the claim line identified as associated with a low-value service. For example, consider a Medicare claim for an individual receiving a low-value arthroscopic lavage for knee osteoarthritis. Claim line 1, day 1 would include a visit code 99213 with a diagnosis code for knee osteoarthritis (ie, M1712); claim line 2, day 2 would include the arthroscopic lavage with a diagnosis code for knee osteoarthritis (ie, M1712); claim line 3, day 2 would also include lactated ringers infusion (ie, J7120), and claim line 4, day 2 would include a fentanyl citrate injection (ie, J3010). Quantifying spending at the claim-level would include spending associated with the arthroscopic lavage procedure, plus the associated services of lactated ringers and fentanyl citrate. Claim line–level spending would include only spending associated with the arthroscopic lavage procedure.

### Statistical Analysis

We quantified per capita use claim line–level and claim-level spending associated with the 32 low-value services. Consistent with prior literature, we reported use and spending per 1000 individuals (for all individuals included in each study year) to provide population-level denominators most relevant for Medicare policy makers.^6,35^ To provide more measure-specific denominators, we also reported the percentage of each service deemed low value by the calculator (among all 32 measures). Specifically, we calculated the number of calculator-designated wasteful services divided by not wasteful services, plus wasteful services, plus likely wasteful services for each measure. We also organized the measures into 5 categories: prescription drugs, diagnostic testing, preventive screening, preoperative testing, and invasive procedures.

We calculated 95% CIs at the measure level under a working assumption of independence between each of the measures. We also adjusted spending trends for inflation using Bureau of Labor data, with 2018 as a reference year.^37^

On October 1, 2015, the Centers for Medicare & Medicaid Services switched from using *ICD-9-CM* to *ICD-10-CM* diagnosis codes, potentially affecting trends of measures using diagnosis codes. To account for this transition, we performed 3 sensitivity analyses assessing use and spending trends among a subset of measures with relatively little reliance on diagnosis codes to assess whether trends differed from our main results. Specifically, we identified (1) 13 study measures in which the measure did not use any diagnosis codes to identify services as not wasteful and the number of *ICD-9-CM* codes was similar to the number of *ICD-10-CM* codes (ie, at least 8 *ICD-9-CM* codes were present for every 10 *ICD-10-CM* codes), (2) 10 measures that did not use any diagnosis codes to identify services as not wasteful, and (3) 8 measures that did not use any diagnosis codes for exclusions or to designate services as not wasteful to determine whether any of these 3 trends analyses differed from our main findings. We also performed sensitivity analyses assessing the potential associations of changing demographic trends and found that they would be highly unlikely to be associated with changes in our results (eAppendix in the Supplement).

All analyses were conducted at RAND Corporation using SAS statistical software version 9.4 (SAS Institute) from September 2019 through December 2020. We explicitly avoided performing statistical testing, considering that these tests would be uniformly significant given the very large sample size reflecting the entire Medicare fee-for-service population, rendering such tests noninformative.

## Results

Among 21 045 759 unique individuals with fee-for-service Medicare, the mean (SD) age was 77.4 (7.9) years and 12 515 915 (59.5%) were women (sex information was missing for 1 individual); 17 846 171 (84.8%) were White individuals, 1 591 659 (7.6%) were Black individuals, 1 273 154 individuals (6.0%) identified as other race/ethnicity, and race/ethnicity information was unknown or missing for 334 775 individuals (1.6%). The number of individuals included in each year increased from 13.4 million in 2014 to 15.4 million in 2018 (Table 1). Mean age (approximately 75 years) and the proportion of women (approximately 60%) remained largely stable throughout the study period.

**Table 1.** Demographic Characteristics

Characteristic	Patients, No. (%)				
	2014 (n = 13 373 850)	2015 (n = 13 892 278)	2016 (n = 14 606 462)	2017 (n = 15 168 134)	2018 (n = 15 364 606)
Age, mean (SD), y	75.7 (7.5)	75.6 (7.5)	75.6 (7.4)	75.3 (7.3)	75.2 (7.3)
Women^a^	8 181 647 (61.2)	8 427 817 (60.7)	8 790 466 (60.2)	9 087 295 (59.9)	9 155 596 (59.6)
Race/ethnicity					
White	11 514 521 (86.1)	12 002 445 (86.4)	12 593 233 (86.2)	12 998 486 (85.7)	13 212 850 (86.0)
Black	982 374 (7.3)	991 175 (7.1)	1 015 617 (7.0)	1 015 630 (6.7)	984 722 (6.4)
Other	792 794 (5.9)	771 152 (5.6)	819 599 (5.6)	866 004 (5.7)	886 983 (5.8)
Unknown or missing race/ethnicity	84 161 (0.63)	127 506 (0.92)	178 013 (1.2)	288 014 (1.9)	280 051 (1.8)

^a^ Among 21 045 759 unique individuals, 1 had missing sex information. This individual was present in the analysis from 2015 to 2018 but not in 2014.

### Use

The percentage of individuals receiving any of the 32 low-value services was 36.3% (95% CI, 36.3%-36.4%) in 2014 and 33.6% (95% CI, 33.6%-33.6%) in 2018. Accordingly, the percentage of individuals receiving not wasteful services was 70.7% (95% CI, 70.7%-70.7%) in 2014 and 71.7% (95% CI, 71.7%-71.7%) in 2018 (Figure).

**Figure.**
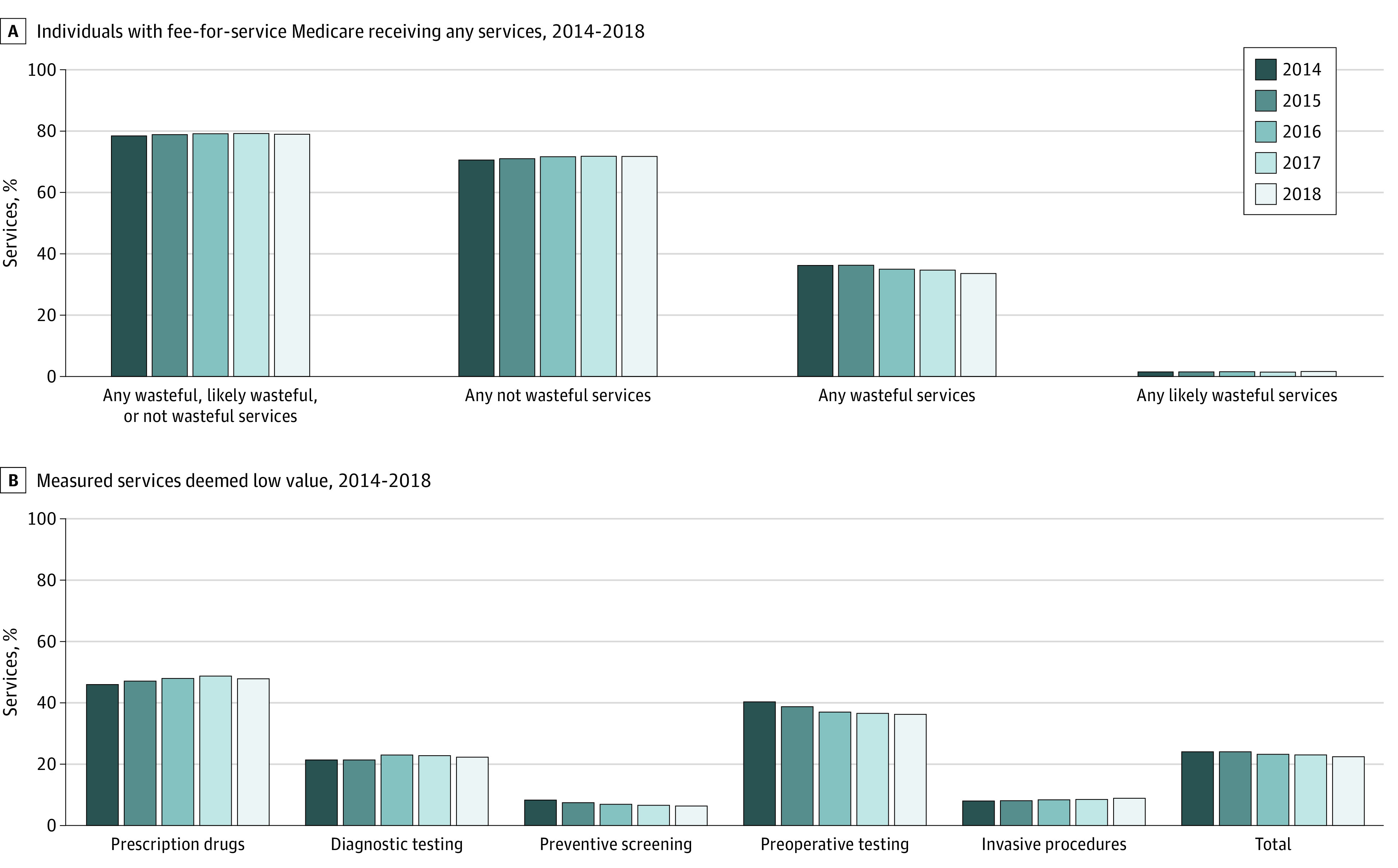
Services Used Among Individuals With Fee-for Service Medicare

Overall, the proportion of individuals receiving 1, 2, 3, 4, or 5 or more low-value services remained stable over time. Uses of low-value service per 1000 individuals with Medicare decreased from 677.8 (95% CI, 676.2-679.5) in 2014 to 632.7 (95% CI, 632.6-632.8) in 2018 (Table 2). Among all measured services, the percentage of services used that were deemed low value decreased from 24.4% (95% CI, 24.4%-24.4%) to 22.7% (95% CI, 22.7%-22.7%) from 2014 to 2018 (Figure).

**Table 2.** Low-Value Care Use

Service	Use per 1000 individuals (95% CI)^a^				
	2014	2015	2016	2017	2018
Total	677.8 (676.2-679.5)	690.6 (689.0-692.1)	662.9 (661.3-664.4)	657.8 (656.2-659.3)	632.7 (632.6-632.8)
Prescription drug	321.3 (319.7-322.9)	362.0 (360.4-363.5)	364.5 (363-366.0)	369.4 (367.9-370.9)	350.4 (350.3-350.4)
Opioid for acute back pain	154.4 (153.6-155.2)	182.4 (181.5-183.2)	187.9 (187-188.7)	187.3 (186.4-188 .l )	182.1 (182.1-182.1)
Oral antibiotic for acute upper respiratory or external ear infections	75.0 (74.9-75.l )	86.7 (86.5-86.8)	84.5 (84.4-84.6)	91.3 (91.2-91.4)	82.0 (82.0-82.0)
Concurrent use of ≥2 antipsychotic medications	32.6 (31.2-33.9)	31.1 (29.8-32.4)	29.6 (28.3-30.8)	29.3 (28.1-30.5)	28.2 (28.1-28.2)
Antidepressant monotherapy in bipolar disorder	0.9.0 (0.9-0.9)	1.0 (0.9-l.0)	0.9 (0.9-0.9)	0.9 (0.9-l.0)	1.0 (0.9-l.0)
NSAID in patient with hypertension, heart failure, or CKD	58.4 (58.2-58.6)	60. 8 (60.6-60.9)	61.5 (61.4-61.7)	60.3 (60.2-60.5)	56.9 (56.9-56.9)
Antibiotic for adenoviral conjunctivitis without secondary infection or other conditions	0.1 (0.1-0.l )	0.1 (0.1-0.1)	0.2 (0.2-0.2)	0.2 (0.2-0.2)	0.2 (0.2-0.2)
Diagnostic test	12.6 (12.5-12.6)	12.5 (12.5-12.6)	13.7 (13.7-13.7)	13.7 (13.7-13.8)	13.5 (13.4-13.6)
Brain imaging for simple syncope with normal neurological exam	0.8 (0.8-0.8)	0.8 (0.8-0.8)	0.9 (0.9-0.9)	0.9 (0.9-0.9)	0.9 (0.9-0.9)
Imaging for uncomplicated headache without neurological symptoms	2.4 (2.3-2.4)	2.2 (2.2-2.2)	2.0 (2.0-2.0)	2.0 (2.0-2.0)	2.0 (2.0-2.0)
Imaging for acute low back pain without red flag signs	1.6 (1.6-l.6)	1.6 (1.6-1.6)	2.1 (2.1-2.l)	2.0 (2.0-2.0)	2.0 (2.0-2.0)
Immunoglobulin G (or immunoglobulin E test in the evaluation of allergy	0.5 (0.5-0.5)	0.5 (0.5-0.5)	0.7 (0.7-0.7)	0.8 (0.8-0.8)	0.8 (0.8-0.9)
Routine diagnostic test for chronic urticaria	0.1 (0.1-0.l)	0.1 (0.1-0.l)	0.1 (0.1-0.l)	0.1 (0.1-0.l)	0.1 (0.1-0.l)
Electroencephalogram for headache	0.5 (0.5-0.5)	0.5 (0.5-0.5)	0.6 (0.6-0.6)	0.6 (0.6-0.6)	0.6 (0.6-0.6)
Carotid duplex ultrasound for simple syncope with normal neurological examination	2.8 (2.8-2.8)	2.9 (2.8-2.9)	3.5 (3.5-3.S)	3.4 (3.4-3.4)	3.2 (3.2-3.2)
CT scan of head or brain for sudden-onset hearing loss	0.6 (0.6-0.6)	0.7 (0.6-0.7)	0.6 (0.6-0.6)	0.7 (0.7-0.7)	0.7 (0.7-0.7)
Imaging for uncomplicated acute rhinosinusitis	1.7 (1.7-1.7)	1.7 (1.7-l.7)	1.7 (1.7-1.7)	1.7 (1.7-l.7)	1.6 (1.6-1.6)
Coronary artery calcium scoring for known CAD	0.1 (0.1-0.l )	0.1 (0.1-0.l )	0.2 (0.1-0.2)	0.2 (0.2-0.2)	0.2 (0.2-0.2)
CT scan for emergency department evaluation of dizziness	1.3 (1.3-1.3)	1.2 (1.2-1.2)	1.2 (1.2-1.2)	1.2 (1.2-l.2)	1.3 (1.3-1.3)
Bleeding time test	0.3 (0.3-0.3)	0.2 (0.2-0.2)	0.1 (0.1-0.l)	0.1 (0.1-0.l)	0.1 (0-0.l)
Preventive screen	118.0 (117.8-118 .l )	107.2 (107.0-107.3)	98.6 (98.5-98.7)	94.l (94.0-94.2)	91.2 (91.2-91.2)
Cervical cancer screen in woman not at high risk with adequate prior screening	24.0 (24-24.l)	21.8 (21.7-21.8)	19.8 (19.8-19.8)	17.9 (17.9-18)	16 .5 (16.5-16.5)
Annual ECG or cardiac screen in patient without symptoms or risk factors	25.5 (25.3-25.6)	21.4 (21.2-21.5)	13.6 (13.5-13.7)	16.9 (16.8-17)	19 .9 (19.9-19.9)
DEXA screen for osteoporosis in woman aged <65 y or man aged <70 y	0.1 (0.1-0.l)	0.1 (0-0.l)	0.1 (0-0.1)	0 (0-0)	0 (0-0)
Unnecessary colorectal cancer screening in adult aged 50-75 y	35.2 (35.2-35.3)	33.2 (33.1-33.2)	32.0 (31.9-32)	31.6 (31.6-31.7)	30.4 (30.4-30.4)
Screen for vitamin D deficiency	26.7 (26.6-26.7)	24.4 (24.3-24.4)	26.4 (26.3-26.4)	21.5 (21.5-21.5)	18.6 (18.6-18.6)
Cardiac stress test or advanced imaging for patient without symptoms or risk factors	6.5 (6.5-6.5)	6.5 (6.4-6.5)	6.8 (6.8-6.8)	6.1 (6.1-6.l)	5.8 (5.8-5.8)
Preoperative test	223.6 (223.2-223.9)	206.3 (206.l-206.6)	183.4 (183.2-183.7)	178 (177.7-178.2)	175.0 (175.0-175.0)
Preoperative laboratory study in patient without significant systemic illness before elective low-risk surgery	213.8 (213.4-214.2)	196.7 (196.4-197)	174.0 (173.7-174.2)	168.7 (168.4-168.9)	166.2 (166.2-166.2)
Preoperative ECG, chest radiograph, or pulmonary function test in patient without significant systemic illness before low-risk surgery	9.5 (9.5-9.5)	9.4 (9.4-9.4)	9.2 (9.2-9.3)	9.0 (9.0-9.0)	8. 6 (8.6-8.6)
Preoperative echocardiogram or stress test before low-risk or intermediate-risk noncardiac surgery	0.2 (0.1-0.2)	0.1 (0.1-0.l)	0.1 (0.1-0.l)	0.1 (0.1-0.l )	0.1 (0.1-0.l)
Routine pulmonary function test before cardiac surgery	0.1 (0.1-0.l )	0.1 (0.1-0.1)	0.1 (0.1-0.1)	0.1 (0.1-0.1)	0.1 (0.1-0.1)
Invasive procedure	2.5 (2.4-2.5)	2.5 (2.5-2.5)	2.6 (2.6-2.6)	2.6 (2.6-2.6)	2.7 (2.6-2.7)
Renal artery revascularization	0.2 (0.2-0.2)	0.2 (0.2-0.2)	0.3 (0.3-0.3)	0.2 (0.2-0.2)	0.3 (0.2-0.3)
Arthroscopic lavage and debridement for knee osteoarthritis	0.1 (0.1-0.l )	0.1 (0.1-0.1)	0.1 (0.l-0.l )	0.1 (0.l-0.l)	0.1 (0.1-0.l )
Peripherally inserted central catheter in patient with stage 3-5 CKD	0.7 (0.7-0.7)	0.8 (0.8-0.8)	0.9 (0.9-0.9)	0.9 (0.9-0.9)	0.9 (0.9-0.9)
Coronary angiogram in patient without symptoms or risk factors	1.4 (1.4-1.4)	1.4 (1.4-1.4)	1.3 (1.3-1.3)	1.4 (1.4-1.4)	1.4 (1.4-1.4)

Abbreviations: CAD, coronary artery disease; CKD, chronic kidney disease; CT, computed tomography; DEXA, dual energy x-ray absorptiometry; ECG, electrocardiogram; NSAID, nonsteroidal anti-inflammatory drug.

^a^ For detailed measure specifications, please see eTable 1 in the Supplement.

Overall, uses of low-value service per 1000 individuals decreased from 677.8 (95% CI, 676.2-679.5) to 632.7 (95% CI, 632.6-632.8) from 2014 to 2018. When assessing trends in uses of low-value services per 1000 individuals within categories of services from 2014 to 2018, preoperative testing decreased from 223.6 (95% CI, 223.2-223.9) to 175.0 (95% CI, 175.0-175.0) (decreasing by 21.7%) and preventive screening decreased from 118.0 (95% CI, 117.8-118.1) to 91.2 (95% CI, 91.2-91.2) (decreasing by 22.7%). In contrast, prescription drugs increased from 321.3 (95% CI, 319.7-322.9) to 350.4 (95% CI, 350.3-350.4) (increasing by 9.1%). Among specific services, 3 services comprised approximately two-thirds of uses among 32 low-value services per 1000 individuals: preoperative laboratory testing decreased from 213.8 (95% CI, 213.4-214.2) to 166.2 (95% CI, 166.2-166.2), opioids for back pain increased from 154.4 (95% CI, 153.6-155.2) to 182.1 (95% CI, 182.1-182.1), and antibiotics for upper respiratory infections increased from 75.0 (95% CI, 75.0-75.1) to 82.0 (95% CI, 82.0-82.0) (Table 2).

### Spending

Spending per 1000 individuals on these 32 low-value services at the line level decreased from $52 765.5 (95% CI, $51 952.3-$53 578.6) to $46 921.7 (95% CI, $46 593.7-$47 249.7) from 2014 to 2018 (Table 3). Spending per 1000 individuals at the claim level also decreased, from $160 070.4 (95% CI, $158 999.8-$161 141.0) to $144 741.1 (95% CI, $144 287.5-$145 194.7) from 2014 to 2018 (Table 4).

**Table 3.** Claim Line–Level Spending

Service	Spending per 1000 individuals, $ (95% CI)^a^				
	2014	2015	2016	2017	2018
Total	52 765.5 (51 952.3-53 578.6)	52 194.4 (51 314.1-53 074.7)	50 783.0 (49 971.8-51 594.3)	47 752.7 (46 951.0-48 554.4)	46 921.7 (46 593.7-47 249.7)
Prescription drug	26 523.0 (25 726.2-27 319.9)	26 222.4 (25 379.0-27 065.9)	23 833.9 (23 056.7-24 611.0)	22 104.0 (21 335.4-22 872.6)	21 175.3 (21 148.7-21 201.9)
Opioid for acute back pain	12 700.0 (12 234.4-13 165.6)	14 540.8 (14 023.9-15 057.7)	14 417.4 (13 946.9-14 888.0)	12 643.1 (12 263.4-13 022.7)	11 884.8 (11 882.7-11 886.9)
Oral antibiotic for acute upper respiratory or external ear infections	1293.5 (1286.5-1300.5)	1314.4 (1308.0-1320.9)	1167.9 (1162.6-1173.2)	1142.4 (1136.0-1148.8)	977.1 (977.0-977.2)
Concurrent use of ≥2 antipsychotic medications	7816.6 (7172.7-8460.5)	7251.8 (6587.4-7916.2)	5975.4 (5358.0-6592.7)	6338.2 (5671.0-7005.5)	6555.4 (6529.7-6581.0)
Antidepressant monotherapy in bipolar disorder	58.0 (52.4-63.5)	52.2 (43.8-60.6)	50.5 (42.5-58.4)	52.5 (42.2-62.8)	50.0 (43.4-56.6)
NSAID in patient with hypertension, heart failure, or CKD	4652.2 (4593.6-4710.8)	3058.7 (3007.5-3109.9)	2216.5 (2180.4-2252.5)	1922.2 (1887.1-1957.2)	1703.8 (1703.3-1704.4)
Antibiotic for adenoviral conjunctivitis without secondary infection or other conditions	2.8 (2.4-3.1)	4.6 (4.1-5.1)	6.2 (5.8-6.6)	5.7 (5.3-6.0)	4.1 (2.8-5.4)
Diagnostic test	2533.2 (2519.1-2547.3)	2454.6 (2441.4-2467.9)	2368.3 (2356.0-2380.6)	2448.1 (2435.9-2460.3)	2443.6 (2417.7-2469.5)
Brain imaging for simple syncope with normal neurological exam	199.0 (195.5-202.5)	212.7 (208.3-217.1)	167 .2 (164-170.4)	163.8 (160.3-167.2)	163.1 (159.6-166.5)
Imaging for uncomplicated headache without neurological symptoms	674.3 (667.1-681.5)	604.5 (597.4-611.7)	528.2 (521.7-534.7)	519.0 (512.7-525.2)	515.2 (512.1-518.2)
Imaging for acute low back pain without red flag signs	169.2 (165.6-172.7)	150.7 (147.8-153.7)	199.3 (195.6-203.0)	189.9 (186.4-193.5)	187.4 (185.5-189.2)
Immunoglobulin G or immunoglobulin E test in the evaluation of allergy	36.5 (35.0-38.0)	39.1 (37.4-40.7)	53.7 (51.7-55.7)	56.6 (54.9-58.3)	54.6 (52.9-56.3)
Routine diagnostic test for chronic urticaria	7.9 (7.0-8.9)	7.2 (6.4-8.1)	10.4 (9.3-11.6)	8.5 (7.7-9.4)	7.2 (1.2-13.1)
Electroencephalogram for headache	279.7 (274.3-285.0)	276.2 (271.2-281.3)	284.7 (279.8-289.5)	289.2 (284.6-293.8)	285.5 (277.5-293.5)
Carotid duplex ultrasound for simple syncope with normal neurological examination	561.0 (558.1-563.8)	594.8 (591.9-597.6)	586.9 (583.6-590.2)	630.5 (627.1-633.9)	624.5 (623.4-625.5)
CT scan of head or brain for sudden-onset hearing loss	101.1 (95.2-107.1)	102.1 (98.4-105.8)	97.4 (94.9-99.9)	110.1 (107.6-112.5)	117.1 (113.8-120.3)
Imaging for uncomplicated acute rhinosinusitis	188.6 (184.8-192.4)	192.4 (187.9-196.8)	191.4 (187.4-195.4)	211.5 (207.3-215.7)	197.7 (195.0-200.4)
Coronary artery calcium scoring for known CAD	33.3 (28.5-38.0)	36.2 (31.9-40.5)	36.3 (32.3-40.3)	51.3 (47.2-55.4)	60.8 (38.0-83.6)
CT scan for emergency department evaluation of dizziness	281.1 (278.0-284.2)	237.7 (234.9-240.4)	212.1 (209.4-214.8)	217.1 (214.4-219.9)	230.3 (228.0-232.7)
Bleeding time test	1.6 (1.4-1.7)	1.1 (1.0-1.3)	0.7 (0.7-0.8)	0.6 (0.5-0.6)	0.4 (−0.3-1.0)
Preventive screen	13 385.5 (13 340.7-13 430.4)	12 971.5 (12 928.9-13 014.1)	12 717.7 (12677.4-12 758.0)	11 575.9 (11 537.0-11 614.8)	11 456.1 (11 449.7-11 462.4)
Cervical cancer screen in woman not at high risk with adequate prior screening	1386.0 (1383.1-1388.9)	1293.3 (1290.6-1296.1)	1187.7 (1185.2-1190.3)	1080.1 (1077.7-1082.4)	939.2 (939.1-939.3)
Annual ECG or cardiac screen in patient without symptoms or risk factors	607.0 (600.3-613.7)	468.7 (464.0-473.5)	311.8 (307.2-316.5)	392.2 (388.2-396.1)	489.7 (488.6-490.8)
DEXA screen for osteoporosis in woman aged <65 y or man aged <70 y	3.5 (3.2-3.8)	2.4 (2.2-2.6)	2.3 (2 .1-2.5)	1.8 (1.6-1.9)	1.9 (−3.3-7.0)
Unnecessary colorectal cancer screening in adult aged 50-75 y	6085.0 (6046.2-6123.8)	5968.7 (5931.6-6005.7)	5748.5 (5713.9-5783.1)	5347.1 (5313.5-5380.8)	5524.8 (5523.3-5526.4)
Screening for vitamin D deficiency	654.3 (650.6-657.9)	640.1 (636.7-643.6)	705.0 (701.8-708.1)	429.5 (426.9-432.2)	358.3 (358.2-358.4)
Cardiac stress test or advanced imaging for patient without symptoms or risk factors	4649.7 (4628.7-4670.7)	4598.2 (4578.1-4618.3)	4762.4 (4742.7-4782.1)	4325.2 (4306.5-4343.9)	4142.2 (4138.9-4145.4)
Preoperative test	4009.5 (3989.8-4029.1)	3761.9 (3737.5-3786.2)	3291.1 (3267.7-3314.4)	3116.2 (3097.4-3135)	3168.6 (3150.3-3186.9)
Preoperative laboratory test in patient without significant systemic illness before elective low-risk surgery	3529.4 (3510.2-3548.7)	3263.6 (3239.6-3287.6)	2874.0 (2850.9-2897.1)	2745.6 (2727.1-2764.2)	2821.8 (2821.7-2821.8)
Preoperative ECG, chest radiograph, or pulmonary function test in patient without significant systemic illness before low-risk surgery	396.8 (394.4-399.1)	429.4 (426.1-432.7)	350.7 (348.3-353.1)	303.8 (301.8-305.9)	282.5 (282.3-282.8)
Preoperative echocardiogram or stress test before low- or intermediate-risk noncardiac surgery	79.5 (76.2-82.8)	67.6 (64.8-70.3)	65.0 (62.7-67.3)	64.9 (62.6-67.2)	62.6 (44.3-80.8)
Routine pulmonary function test before cardiac surgery	3.8 (3.5-4.0)	1.3 (1.2-1.5)	1.3 (1.2-1.5)	1.8 (1.6-2.0)	1.8 (0.3-3.3)
Invasive procedure	6314.2 (6160.2-6468.3)	6783.9 (6536.9-7030.9)	8572.1 (8344.2-8800.1)	8508.5 (8284.7-8732.2)	8678.1 (8352.8-9003.4)
Renal artery revascularization	1086.8 (1025.7-1147.9)	1261.1 (1176.8-1345.3)	1532.2 (1476.4-1588.0)	1453.9 (1385.6-1522.1)	1582.9 (1375.1-1790.6)
Arthroscopic lavage and debridement for knee osteoarthritis	149.6 (143-156.2)	152.4 (146.3-158.6)	146.2 (139.7-152.7)	126.8 (121.3-132.4)	124.7 (58.8-190.6)
Peripherally inserted central catheter in patient with stage 3-5 CKD	1610.2 (1475.0-1745.4)	2252.4 (2022.9-2481.9)	3940.8 (3722.6-4159.1)	3626.2 (3416.7-3835.7)	3596.0 (3356.2-3835.9)
Coronary angiogram in patients without symptoms or risk factors	3467.7 (3426.9-3508.5)	3118 (3083.3-3152.7)	2952.9 (2918.5-2987.2)	3301.6 (3263.0-3340.3)	3374.5 (3346.3-3402.7)

Abbreviations: CAD, coronary artery disease; CKD, chronic kidney disease; CT, computed tomography; DEXA, dual energy x-ray absorptiometry; ECG, electrocardiogram; NSAID, nonsteroidal anti-inflammatory drug.

^a^ For detailed measure specifications, please see eTable 1 in the Supplement.

**Table 4.** Claim-Level Spending

Service	Spending per 1000 individuals, $ (95% CI)^a^				
	2014	2015	2016	2017	2018
Total	160 070.4 (158 999.8-161 141.0)	160 321.3 (156 308.0-164 334.7)	145 722.7 (144 503.2-146 942.2)	141 873.7 (140 601.3-143 146.0)	144 741.1 (144 287.5-145 194.7)
Prescription drug	26 523.4 (25 726.5-27 320.2)	29 641.6 (28 519.0-30 764.3)	26 830.3 (25 783.2-27 877.5)	25 314.2 (24 223.3-26 405.1)	24 532.0 (24 491.5-24 572.5)
Opioid for acute back pain	12 700.0 (12 234.4-13 165.6)	14 540.4 (14 023.5-15 057.3)	14 417.7 (13 947.2-14 888.3)	12 642.9 (12 263.2-13022 .5)	11 885 (11 882.9, 11 887.1)
Oral antibiotic for acute upper respiratory or external ear infections	1293.4 (1286.5-1300.4)	1314.5 (1308.0-1320.9)	1167.9 (1162.6-1173.2)	1142.3 (1136.0-1148.7)	977.l (977.0-977.2)
Concurrent use of ≥2 or more antipsychotic medications	7816.9 (7173.0-8460.8)	10671.3 (9676.1-11 666.4)	8971.6 (8036.9-9906.3)	9548.7 (8526.7-10 570.7)	9912.0 (9872.1-9951.9)
Antidepressant monotherapy in bipolar disorder	58.0 (52.4-63.5)	52.2 (43.8-60.6)	50.5 (42.5, 58.4)	52.5 (42.2-62.8)	50.0 (43.4-56.6)
NSAID in patient with hypertension, heart failure, or CKD	4652.3 (4593.6-4710.9)	3058.7 (3007.5-3109.9)	2216.5 (2180.4-2252.5)	1922.1 (1887.1-1957.2)	1703.8 (1703.2-1704.4)
Antibiotic for adenoviral conjunctivitis without secondary infection or other conditions	2.8 (2.4-3.1)	4.6 (4.1-5.1)	6.2 (5.8-6.6)	5.7 (5.3-6.0)	4.1 (2.8-5.4)
Diagnostic test	7824.3 (7703.4-7945.2)	7649.8 (7580.6-7719.0)	7855.2 (7794.0-7916.3)	7939.8 (7887.0-7992.6)	7951.9 (7721.1-8182.6)
Brain imaging for simple syncope with normal neurological exam	1040.8 (1020.4-1061.1)	1123.4 (1102.3-1144.5)	1209.1 (1190.4-1227.7)	1192.1 (1173.3-1211.0)	1227.0 (1206.2-1247.8)
Imaging for uncomplicated headache without neurological symptoms	1368.4 (1351.9-1384.9)	1265.1 (1249.6-1280.6)	1156.1 (1141.4-1170.9)	1171.6 (1157.9-1185.4)	1175.9 (1168.7-1183.1)
Imaging for acute low back pain without red flag signs	372.5 (364.6-380.4)	356.7 (349.7-363.8)	453.6 (445.2-462.0)	430.5 (422.7-438.4)	426.5 (422.3-430.7)
Immunoglobulin G or immunoglobulin E test in the evaluation of allergy	103.5 (91.7, 115.2)	106.1 (91.6-120.7)	150.5 (119.8-181.3)	165.8 (150.4-181.2)	160.2 (144.2-176.1)
Routine diagnostic test for chronic urticaria	29.9 (23.5-36.4)	36.1 (25.1-47.0)	35.8 (28.4-43.1)	47.8 (39.4-56.3)	44.4 (−41.1-129.9)
Electroencephalogram for headache	410.4 (399.3-421.4)	402.8 (392.5-413.1)	413.1 (403.5-422.7)	427.0 (416.9-437.2)	424.0 (403.4-444.6)
Carotid duplex ultrasound for simple syncope with normal neurological examination	2090.9 (2057.4-2124.3)	2162.6 (2130.4-2194.7)	2362.8 (2331.8-2393.9)	2272.2 (2243.8-2300.7)	2216.5 (2207.6-2225.4)
CT scan of head or brain for sudden onset hearing loss	256.8 (152.5-361.1)	232.1 (198.2-266.0)	212.2 (193.2-231.2)	226.6 (216.8-236.3)	247.9 (231.9-263.8)
Imaging for uncomplicated acute rhinosinusitis	595.3 (582.1-608.5)	600.1 (586.4-613.8)	623.5 (608.7-638.2)	684.3 (669.2-699.4)	632.4 (623.3-641.5)
Coronary artery calcium scoring for known CAD	38.9 (34.1-43.7)	43.1 (38.7-47.4)	44.3 (40.3-48.4)	62.1 (57.9-66.2)	72.7 (49.6-95.7)
CT scan for emergency department evaluation of dizziness	1198.1 (1183.1-1213.0)	1070.6 (1056.4-1084.9)	1070.0 (1056.6-1083.4)	1138.4 (1126.2-1150.5)	1217.3 (1207.2-1227.4)
Bleeding time test	318.9 (285.0-352.8)	251.1 (219.1-283.0)	124.1 (107.4,-140.8)	121.3 (99.6-143.1)	107.2 (−101.9-316.2)
Preventive screen	29 841.2 (29 431.5-30 250.9)	28 106 (27 787.1-28 424.9)	26 129.1 (25 884.9-26 373.3)	24 786.3 (24 529.5-25 043.0)	24 413.2 (24 391.8-24 434.7)
Cervical cancer screen in woman not at high risk with adequate prior screening	2721.2 (2711.8-2730.6)	2555.7 (2546.3-2565.2)	2345.5 (2336.1-2354.9)	2138.8 (2130.1-2147.4)	1914.8 (1914.2-1915.4)
Annual ECG or cardiac screen in patient without symptoms or risk factors	6453.8 (6073.4-6834.1)	5681.4 (5386.9-5975.9)	4121.0 (3895.7-4346.4)	4609.1 (4373.7-4844.5)	5062.8 (5049.5-5076.1)
DEXA screen for osteoporosis in woman aged <65 y or man aged <70y	7.4 (5.5-9.2)	5.5 (4.7-6.2)	5.3 (4.1-6.5)	3.1 (2.8-3.5)	3.2 (−10.6-17.1)
Unnecessary colorectal cancer screen in adult aged 50-75 y	11 057 (10 919.5-11 194.5)	10 634.5 (10 541.5-10 727.4)	9995.9 (9927.8-10 064.1)	9589.2 (9510.8-9667.6)	9593 (9587.2-9598.8)
Screen for vitamin D deficiency	3702 (3658.2-3745.9)	3435.3 (3369.9-3500.7)	3659.2 (3606.1-3712.3)	2962.6 (2906.7-3018.4)	2639.9 (2636.9-2642.9)
Cardiac stress test or advanced imaging for patient without symptoms or risk factors	5899.9 (5852.6-5947.2)	5793.6 (5749.4-5837.9)	6002.1 (5966.3-6037.9)	5483.6 (5449.2-5518.0)	5199.5 (5192.4-5206.6)
Preoperative test	85 993.0 (85 450.1-86 536.0)	82 330.5 (81 777.0-82 883.9)	72 393.8 (71 883.3-72 904.3)	71 365.4 (70 824.1-71 906.6)	74 585.1 (74 502.6-74 667.6)
Preoperative laboratory study in patient without significant systemic illness before elective low-risk surgery	79 184.9 (78 656.2-79 713.7)	76 023.4 (75 476.6-76 570.2)	66 718.8 (66 214.0-67 223.5)	65 908.2 (65 372.0-66 444.3)	69 127.0 (69 123.8-69 130.2)
Preoperative ECG, chest radiograph, or pulmonary function test in patient without significant systemic illness before low-risk surgery	6635.3 (6512.3-6758.2)	6155.3 (6070.6-6240)	5509.5 (5434.4-5584.6)	5277.0 (5203.8-5350.3)	5286.7 (5278.0-5295.4)
Preoperative echocardiogram or stress test before low-risk or intermediate-risk noncardiac surgery	114.3 (104.3-124.4)	89.6 (83.9-95.3)	92.6 (83.2-102.1)	87.5 (82.6-92.4)	85.0 (39.4-130.7)
Routine pulmonary function test before cardiac surgery	58.5 (51.7- 65.4)	62.2 (54 .6-69.7)	72.9 (64.8-81.1)	92.6 (83.0-102.3)	86.4 (18.3-154.5)
Invasive procedure	9888.5 (9704.1-10 072.9)	12 593.5 (8 794.3, 16392 .7)	12514.3 (12 256.1-12 772.5)	12 468.0 (12 208.8-12 727.3)	13 258.8 (12 879.8-13 637.8)
Renal artery revascularization	1486.1 (1415.8-1556.4)	1591.7 (1504.7-1678.7)	1973.6 (1907.8-2039.5)	1776.7 (1703.2-1850.3)	1937.6 (1718.7-2156.5)
Arthroscopic lavage and debridement for knee osteoarthritis	149.6 (143.0-156.2)	152.4 (146.3-158.6)	146.2 (139.7-152.7)	126.8 (121.3-132.4)	124.7 (58.8-190.6)
Peripherally inserted central catheter in patient with stage 3-5 CKD	2692.5 (2546.7-2838.3)	5482.4 (1685.1-9279.8)	5501.1 (5261.6-5740.6)	5379.4 (5141.0-5617.7)	5797.8 (5500.2-6095.4)
Coronary angiogram in patient without symptoms or risk factors	5560.3 (5472.3-5648.4)	5366.9 (5286.2-5447.7)	4893.3 (4823.1-4963.6)	5185.1 (5114.7-5255.5)	5398.7 (5345.5-5451.9)

Abbreviations: CAD, coronary artery disease; CKD, chronic kidney disease; CT, computed tomography; DEXA, dual energy x-ray absorptiometry; ECG, electrocardiogram; NSAID, nonsteroidal anti-inflammatory drug.

^a^ For detailed measure specifications, please see eTable 1 in the Supplement.

From 2014 to 2018, within low-value service categories, spending per 1000 individuals on preoperative testing decreased from $4009.5 (95% CI, $3989.8-$4029.1) to $3168.6 (95% CI, $3150.3-$3186.9) (decreasing by 21.0%) at the claim-line-level and from $85 993.0 (95% CI, $85 450.1-$86 536.0) to $74 585.1 (95% CI, $74 502.6-$74 667.6) (decreasing by 13.3%) at the claim level. In contrast, spending per 1000 individuals on invasive procedures increased from $6314.2 (95% CI, $6160.2-6468.3) to $8678.1 (95% CI, $8352.8-$9003.4 ) (increasing by 37.4%) at the claim-line level and from $9888.5 (95% CI, $9704.1-$10 072.9) to $13 258.8 (95% CI, $12 879.8-$13 637.8) (increasing by 34.1%) at the claim level (Table 3 and Table 4). As use remained stable overall for invasive procedures, this increase in spending was partly associated with the increases in claim-level and claim line–level unit spending for peripherally inserted central catheters (PICCs).

### Sensitivity Analyses

Compared with our main findings, we found similar results when assessing use and spending trends among the (1) 13 measures that did not use any diagnosis codes to classify not wasteful services or used a similar number of *ICD-9-CM* and *ICD-10-CM* codes to classify not wasteful services, (2) 10 measures with no *ICD-9-CM* or *ICD-10-CM* codes used to classify not wasteful services, and (3) 8 measures that did not use any diagnosis codes for exclusions or for determining whether a service was not wasteful (eTable 2 in the Supplement). The analyses found decreases in use and claim line–level spending and no change in claim-level spending, with the exception that claim-level spending increased for the analysis including 8 measures.

## Discussion

This cross-sectional study found that among 32 measured services, low-value care use and spending levels decreased marginally from 2014 to 2018 among individuals with fee-for-service Medicare, despite a national education campaign in collaboration with clinician specialty societies and increased use of payment models that hold clinicians accountable for total cost of care.^19^ Three items comprised the bulk of low-value services. Of these, opioid prescriptions increased, despite the documented patient harms associated with these services. As the Medicare program continues to face mounting financial pressures, our study highlights several important opportunities for targeted interventions that may reduce wasteful spending while improving the quality of care.

Although the measures, timing, and population in our study differ from those in prior work, our results are generally consistent with those of other studies evaluating trends in low-value care. Two trends analyses from 2017^24,25,26,27,28,29,30,33,34,35^ and 1 from 2015^26^ found that overall low-value care use and spending remained similar or declined slightly over time after the advent of the Choosing Wisely Campaign; however, these studies used data from 2009 to 2015, assessed claims-based measures related to but different from ours, and focused on individuals with commercial insurance and Medicare Advantage. Moreover, the Choosing Wisely campaign had not expanded yet to more measures, and it took time for awareness of the program to increase; hence, our 2014 to 2018 time frame has allowed more time to observe potential changes. Our findings are also broadly consistent with those of the 2020 Health Care Cost Institute report^38^ on overall use and spending trends, which suggest that increases in unit prices, rather than use, are the main factor associated with rising US health care spending from 2014 to 2018. Moreover, while use of preoperative testing and preventive services decreased over time in our study, use of many services seems to be continuing to increase, which raises important questions. Is the evidence less agreed on for those services? Have there been few interventions in those spaces? Is there more clinical uncertainty about what is recommended under clinical scenarios, so physicians may err on the side of being more cautious?

As we considered these questions, our findings of increased low-value antibiotic prescribing among older adults with acute upper respiratory infections were particularly notable in light of strong evidence demonstrating lack of benefit of these treatments and increasing overall attention on antibiotic stewardship and appropriate antibiotic prescribing.^39,40,41,42,43^ Nevertheless, these findings are consistent with those of a 2020 study^44^ using pharmaceutical data for overall antibiotic prescribing within a subgroup of older adults from 2011 to 2016. Studies from 2016^45^ and 2020^46^ found that physicians who feel rushed or do not believe that antibiotics are overused are more likely to prescribe low-value antibiotics, while patients often desire antibiotic prescriptions for upper respiratory infections despite knowledge that these medications are overprescribed and contribute to antibiotic resistance. Evidence from a 2017 review^47^ and a 2016 randomized clinical trial^41^ suggests that electronic health record–based clinical decision support (EHR CDS) tools applying behavioral economics may help alleviate this problem. A 2016 cluster randomized clinical trial^48^ found that clinician education strategies alone helped initially, but their effects waned over time, whereas EHR CDS tools providing real-time decision support to ordering clinicians showed longer-term effectiveness.

We noted an increase in opioid prescriptions for acute low back pain, results that differed from those of other published studies (including our own work); a 2019 study^49^ and a 2018 study^50^ found that overall opioid prescribing decreased among US ambulatory visits from 2014 to 2016 and did not change among individuals with commercial insurance or Medicare Advantage from 2014 to 2017.^49,51^ Important differences in our measure were that we focused on acute back pain and included a distinct population from prior work, specifically, individuals continuously enrolled in fee-for-service Medicare. The previous 2 studies assessed overall initial prescribing and assessed either commercial insurance and Medicare Advantage populations or ambulatory visit data representing all US adults. Systematic reviews from 2020 and 2019 of interventions to address this obstinate problem^50,52^ found that while prescription monitoring programs were associated with a successful reduction in overall opioid prescribing, their association with the appropriate use of these prescriptions remains as yet unknown. To be sure, while increasing antibiotic and opioid prescriptions was associated with the increase in prescription drug use, the overall prescription drug spending decrease was partly associated with decreases in spending on nonsteroidal anti-inflammatory drugs, potentially associated in part with Celebrex (celecoxib) becoming generic in mid-2014. Nevertheless, in the midst of ongoing antibiotic overuse and an opioid overdose crisis,^43,53^ our findings highlight worrisome trends and underscore an urgent need to improve the quality and safety of care delivered to individuals with Medicare.

While educational efforts, such as the Choosing Wisely campaign, are important for raising awareness of the problem among clinicians and patients, additional efforts will be needed to significantly curb low-value care use and spending in light of our findings.^54,55^ Specifically, we found that increases in the price of services, such as certain diagnostic imaging tests and invasive procedures (eg, PICCs), were also associated with increases in low-value care spending, and addressing price increases may represent an important strategy in reducing wasteful spending.^38^ In terms of use, capitated payment models and accountable care organizations (ACOs) comprise an important strategy that may incentivize clinicians to reduce low-value care use across a broad range of clinical arenas. A 2015 study^56^ found that participation in the Medicare Pioneer ACO program was associated with decreases in use of low-value services. While expanding and strengthening capitated and ACO payment models may be associated with larger changes in physician behavior, policy makers must also carefully monitor for unintended harms, such as decreases in use of high-value care.^1,2,57,58^ On the demand side, encouraging use of shared decision-making and aligning benefit design with payment reform could also be associated with incremental success, particularly when carefully targeting cost sharing exclusively toward low-value care.^59,60,61,62^ To be sure, financial incentives are necessary but likely insufficient by themselves,^1,8,63,64^ considering that a 2017 systematic review^47^ of interventions to reduce low-value care found that multicomponent interventions are associated with superior outcomes compared with single-component interventions. Most likely, a combination of capitated payment models combined with physician engagement and culture change, alongside the implementation of seamless EHR CDS tools, may have the greatest chance of success in eliminating low-value care.^1,17,63,65^

### Limitations

This study has several limitations. First, findings reflect trends among individuals with fee-for-service Medicare and may not generalize among other populations. Second, this analysis reflects only the narrow segment of low-value care that has broad professional consensus and is readily amenable to claims-based measurement over 5 years’ time; trends may differ among unmeasured potentially low-value services. Third, the low-value care measure algorithms are proprietary; however, our appendix outlines detailed measure specifications. Fourth, all claims-based analyses are inherently limited in terms of clinical validity. They may underestimate or overestimate true low-value care prevalence, as claims do not fully reflect the clinical circumstances that make a given service high value or low value. While it is unlikely that the rate of low-value care measurement misclassification error has changed over time, a 2019 study^66^ comparing analogous Choosing Wisely claims-based low-value care measures reported a median 80% sensitivity and 88% specificity compared with professional medical record review. Fifth, the diagnosis code transition from *ICD-9-CM* to *ICD-10-CM* may be associated with biases in our results. However, our sensitivity analyses using measures associated minimally with diagnosis codes were consistent with our overall findings. Moreover, use continued to marginally decline 3 years in a row after the *ICD-9-CM* to *ICD-10-CM* transition, from 2016 to 2018. Nevertheless, we cannot fully eliminate the possibility that the diagnosis code transition is associated with changes to our results. Sixth, while our denominators are at the total beneficiary level and service level, they are not clinically targeted measures (eg, for the preoperative testing measures, clinically targeted measures would include individuals undergoing low-risk surgeries). Nevertheless, we found stable trends among not wasteful services, which suggests that targeted clinical denominators are likely stable over time. Seventh, we used 2 methods for estimating spending because the claim line–level spending count method may miss spending associated with a low-value service if that spending is represented in another claim line or revenue center on the claim. This is associated with underestimation of spending on low-value care. The claim-level spending count method may include services not associated with the low-value service but billed on the same claim, which is associated with overestimation of the cost of a low-value service. Moreover, associated services billed on a different claim from the low-value service itself will not be captured.

## Conclusions

This cross-sectional study found that among individuals with fee-for-service Medicare receiving any of 32 measured services, low-value care use and spending decreased marginally from 2014 to 2018, despite a national education campaign in collaboration with clinician specialty societies and increased attention on low-value care. Three items comprised the bulk of low-value services. Of these, opioid prescriptions increased over time, despite the documented patient harms associated with these services. As the US continues to grapple with the financial sustainability of the Medicare program, our findings highlight several important opportunities that may help decrease use of low-value care and inform the design of interventions that avoid blunt cost reductions by specifically targeting wasteful spending.

## Notes

Supplement. **eAppendix.** Sensitivity Analyses on the Impact of Demographic Trends **eFigure.** Study Design: 5 Repeated Cross-Sectional Cohorts, 2014-2018 **eTable 1.** Milliman MedInsight Health Waste Calculator Specifications for 32 Measures of Low-Value Care **eTable 2.** Three Sensitivity Analyses on Trends Including Measures With Relatively Little Reliance on Diagnosis Codes

## References

[1] Mafi JN, Parchman M Low-value care: an intractable global problem with no quick fix. BMJ Qual Saf. 2018;27(5):333-336. doi:10.1136/bmjqs-2017-007477 29331955 PMC6727656

[2] Kerr EA, Kullgren JT, Saini SD Choosing wisely: how to fulfill the promise in the next 5 years. Health Aff (Millwood). 2017;36(11):2012-2018. doi:10.1377/hlthaff.2017.0953 29137505

[3] Institute of Medicine Best Care at Lower Cost: The Path to Continuously Learning Health Care in America. The National Academies Press; 2013. 24901184

[4] Berwick DM, Hackbarth AD Eliminating waste in US health care. JAMA. 2012;307(14):1513-1516. doi:10.1001/jama.2012.362 22419800

[5] Shehab N, Lovegrove MC, Geller AI, Rose KO, Weidle NJ, Budnitz DS US emergency department visits for outpatient adverse drug events, 2013-2014. JAMA. 2016;316(20):2115-2125. doi:10.1001/jama.2016.16201 27893129 PMC6490178

[6] Schwartz AL, Landon BE, Elshaug AG, Chernew ME, McWilliams JM Measuring low-value care in Medicare. JAMA Intern Med. 2014;174(7):1067-1076. doi:10.1001/jamainternmed.2014.1541 24819824 PMC4241845

[7] Mafi JN, Russell K, Bortz BA, Dachary M, Hazel WA Jr, Fendrick AM Low-cost, high-volume health services contribute the most to unnecessary health spending. Health Aff (Millwood). 2017;36(10):1701-1704. doi:10.1377/hlthaff.2017.0385 28971913 PMC6727655

[8] Barnett ML, Linder JA, Clark CR, Sommers BD Low-value medical services in the safety-net population. JAMA Intern Med. 2017;177(6):829-837. doi:10.1001/jamainternmed.2017.0401 28395014 PMC5540058

[9] Mafi JN, Wee CC, Davis RB, Landon BE Association of primary care practice location and ownership with the provision of low-value care in the United States. JAMA Intern Med. 2017;177(6):838-845. doi:10.1001/jamainternmed.2017.0410 28395013 PMC5540052

[10] Colla CH, Morden NE, Sequist TD, Schpero WL, Rosenthal MB Choosing wisely: prevalence and correlates of low-value health care services in the United States. J Gen Intern Med. 2015;30(2):221-228. doi:10.1007/s11606-014-3070-z 25373832 PMC4314495

[11] Mafi JN, May FP, Kahn KL, Low-value proton pump inhibitor prescriptions among older adults at a large academic health system. J Am Geriatr Soc. 2019;67(12):2600-2604. doi:10.1111/jgs.16117 31486549 PMC6952216

[12] Mafi JN, Wee CC, Davis RB, Landon BE comparing use of low-value health care services among U.S. advanced practice clinicians and physicians. Ann Intern Med. 2016;165(4):237-244. doi:10.7326/M15-2152 27322541 PMC5584613

[13] Mafi JN, Edwards ST, Pedersen NP, Davis RB, McCarthy EP, Landon BE Trends in the ambulatory management of headache: analysis of NAMCS and NHAMCS data 1999-2010. J Gen Intern Med. 2015;30(5):548-555. doi:10.1007/s11606-014-3107-3 25567755 PMC4395605

[14] Mafi JN, McCarthy EP, Davis RB, Landon BE Worsening trends in the management and treatment of back pain. JAMA Intern Med. 2013;173(17):1573-1581. doi:10.1001/jamainternmed.2013.8992 23896698 PMC4381435

[15] Reid RO, Rabideau B, Sood N Low-value health care services in a commercially insured population. JAMA Intern Med. 2016;176(10):1567-1571. doi:10.1001/jamainternmed.2016.5031 27571327

[16] MedPAC Use of low-value care in Medicare is substantial. MedPAC Blog. Accessed June 4, 2020. http://www.medpac.gov/-blog-/medpacblog/2015/05/21/use-of-low-value-care-in-medicare-is-substantial

[17] Damberg CL, Silverman M, Burgette L, Vaiana ME, Ridgely MS Are value-based incentives driving behavior change to improve value? Am J Manag Care. 2019;25(2):e26-e32. 30763040 PMC8502100

[18] Haverkamp MH, Peiris D, Mainor AJ, ACOs with risk-bearing experience are likely taking steps to reduce low-value medical services. Am J Manag Care. 2018;24(7):e216-e221. 30020757 PMC6594369

[19] McWilliams JM, Landon BE, Rathi VK, Chernew ME Getting more savings from ACOs — can the pace be pushed? N Engl J Med. 2019;380(23):2190-2192. doi:10.1056/NEJMp1900537 31167048 PMC6624070

[20] McWilliams JM, Chernew ME, Landon BE, Schwartz AL Performance differences in year 1 of Pioneer accountable care organizations. N Engl J Med. 2015;372(20):1927-1936. doi:10.1056/NEJMsa1414929 25875195 PMC4475634

[21] Roberts ET, McWilliams JM, Hatfield LA, Changes in health care use associated with the introduction of hospital global budgets in Maryland. JAMA Intern Med. 2018;178(2):260-268. doi:10.1001/jamainternmed.2017.7455 29340564 PMC5838791

[22] Roberts ET, Hatfield LA, McWilliams JM, Changes in hospital utilization three years into Maryland’s global budget program for rural hospitals. Health Aff (Millwood). 2018;37(4):644-653. doi:10.1377/hlthaff.2018.0112 29608370 PMC5993431

[23] Segal JB, Bridges JF, Chang HY, Identifying possible indicators of systematic overuse of health care procedures with claims data. Med Care. 2014;52(2):157-163. doi:10.1097/MLR.0000000000000052 24374418

[24] Hong AS, Ross-Degnan D, Zhang F, Wharam JF Small decline in low-value back imaging associated with the ‘Choosing Wisely’ campaign, 2012-14. Health Aff (Millwood). 2017;36(4):671-679. doi:10.1377/hlthaff.2016.1263 28373333

[25] Carter EA, Morin PE, Lind KD Costs and trends in utilization of low-value services among older adults with commercial insurance or Medicare Advantage. Med Care. 2017;55(11):931-939. doi:10.1097/MLR.0000000000000809 28930892

[26] Rosenberg A, Agiro A, Gottlieb M, Early trends among seven recommendations from the Choosing Wisely campaign. JAMA Intern Med. 2015;175(12):1913-1920. doi:10.1001/jamainternmed.2015.5441 26457643

[27] Oakes AH, Chang HY, Segal JB Systemic overuse of health care in a commercially insured US population, 2010-2015. BMC Health Serv Res. 2019;19(1):280. doi:10.1186/s12913-019-4079-0 31046746 PMC6498548

[28] Reid R, Damberg C, Friedberg MW Primary care spending in the fee-for-service Medicare population. JAMA Intern Med. 2019;179(7):977-980. doi:10.1001/jamainternmed.2018.8747 30985864 PMC6583869

[29] Milliman MedInsight health waste calculator. Accessed February 20, 2020. https://milliman-cdn.azureedge.net/-/media/medinsight/pdfs/medinsight-health-waste-calculator.ashx

[30] Brown DL, Clement F Calculating health care waste in Washington state: first, do no harm. JAMA Intern Med. 2018;178(9):1262-1263. doi:10.1001/jamainternmed.2018.3516 30083773

[31] US Centers for Disease Control and Prevention *International Classification of Diseases, Ninth Revision, Clinical Modification (ICD-9-CM)* Accessed January 15, 2021. https://www.cdc.gov/nchs/icd/icd9cm.htm

[32] US Centers for Disease Control and Prevention *International Classification of Diseases, Tenth Revision, Clinical Modification (ICD-10-CM)* Accessed January 12, 2021. https://www.cdc.gov/nchs/icd/icd10cm.htm

[33] Virginia Health Information Virginia APCD MedInsight health waste calculator results version 2.0. Accessed June 2, 2017. http://www.vahealthinnovation.org/wp-content/uploads/2016/10/Virginia-APCD-MedInsight-Health-Waste-Calculator-Results-v2.0.pdf

[34] Washington Health Alliance. Drop the pre-op! Accessed April 18, 2019. https://wahealthalliance.org/wp-content/uploads/2018/10/Drop-the-Pre-op-Info-Sheet-09.14.pdf

[35] Reid RO, Mafi JN, Baseman LH, Fendrick AM, Damberg CL Waste in the Medicare program: a national cross-sectional analysis of 2017 low-value service use and spending. J Gen Intern Med. 2020. doi:10.1007/s11606-020-06061-0 32728953 PMC8342744

[36] Research Data Assistance Center Definitions of ‘cost’ in Medicare utilization files. Accessed May 26, 2020. https://www.resdac.org/videos/definitions-cost-medicare-utilization-files

[37] US Bureau of Labor Statistics Consumer Price Index inflation calculator. Accessed November 24, 2020. https://www.bls.gov/data/inflation_calculator.htm

[38] Health Care Cost Institute 2018 Health care cost and utilization report. Accessed January 7, 2020. https://healthcostinstitute.org/images/pdfs/HCCI_2018_Health_Care_Cost_and_Utilization_Report.pdf

[39] Chua K-P, Fischer MA, Linder JA Appropriateness of outpatient antibiotic prescribing among privately insured US patients: *ICD-10-CM* based cross sectional study. BMJ. 2019;364:k5092-k5092. doi:10.1136/bmj.k5092 30651273 PMC6334180

[40] Gjelstad S, Høye S, Straand J, Brekke M, Dalen I, Lindbæk M Improving antibiotic prescribing in acute respiratory tract infections: cluster randomised trial from Norwegian general practice (Prescription Peer Academic Detailing (Rx-PAD) study). BMJ. 2013;347:f4403. doi:10.1136/bmj.f4403 23894178 PMC3724398

[41] Meeker D, Linder JA, Fox CR, Effect of behavioral interventions on inappropriate antibiotic prescribing among primary care practices: a randomized clinical trial. JAMA. 2016;315(6):562-570. doi:10.1001/jama.2016.0275 26864410 PMC6689234

[42] Barnett ML, Linder JA Antibiotic prescribing to adults with sore throat in the United States, 1997-2010. JAMA Intern Med. 2014;174(1):138-140. doi:10.1001/jamainternmed.2013.11673 24091806 PMC4526245

[43] US Centers for Disease Control and Prevention Measuring outpatient antibiotic prescribing. Accessed November 30, 2020. https://www.cdc.gov/antibiotic-use/community/programs-measurement/measuring-antibiotic-prescribing.html#annual-report

[44] King LM, Bartoces M, Fleming-Dutra KE, Roberts RM, Hicks LA Changes in US outpatient antibiotic prescriptions from 2011-2016. Clin Infect Dis. 2020;70(3):370-377. 30882145 10.1093/cid/ciz225 PMC8078491

[45] Gidengil CA, Mehrotra A, Beach S, Setodji C, Hunter G, Linder JA What drives variation in antibiotic prescribing for acute respiratory infections? J Gen Intern Med. 2016;31(8):918-924. doi:10.1007/s11606-016-3643-0 27067351 PMC4945551

[46] Cuevas MA, Wachter ND, Reyes C, Seeking care for back pain or upper respiratory infections: survey results to inform a safety net hospital Choosing Wisely intervention. Healthc (Amst). 2020;8(3):100424. doi:10.1016/j.hjdsi.2020.100424 32919578 PMC7490460

[47] Colla CH, Mainor AJ, Hargreaves C, Sequist T, Morden N Interventions aimed at reducing use of low-value health services: a systematic review. Med Care Res Rev. 2017;74(5):507-550. doi:10.1177/1077558716656970 27402662

[48] Sharp AL, Hu YR, Shen E, Improving antibiotic stewardship: a stepped-wedge cluster randomized trial. Am J Manag Care. 2017;23(11):e360-e365. 29182356

[49] Zhu W, Chernew ME, Sherry TB, Maestas N Initial opioid prescriptions among U.S. commercially insured patients, 2012-2017. N Engl J Med. 2019;380(11):1043-1052. doi:10.1056/NEJMsa1807069 30865798 PMC6487883

[50] Awadalla R, Gnjidic D, Patanwala A, Sakiris M, Penm J The effectiveness of stewardship interventions to reduce the prescribing of extended-release opioids for acute pain: a systematic review. Pain Med. 2020;21(10):2401-2411. doi:10.1093/pm/pnaa139 32488237

[51] Ladapo JA, Larochelle MR, Chen A, Physician prescribing of opioids to patients at increased risk of overdose from benzodiazepine use in the United States. JAMA Psychiatry. 2018;75(6):623-630. doi:10.1001/jamapsychiatry.2018.0544 29710086 PMC6137520

[52] Moride Y, Lemieux-Uresandi D, Castillon G, A systematic review of interventions and programs targeting appropriate prescribing of opioids. Pain Physician. 2019;22(3):229-240. 31151331

[53] US Centers for Disease Control and Prevention Prescription Opioid Data. Accessed November 30, 2020. https://www.cdc.gov/drugoverdose/data/prescribing.html

[54] Rich EC Barriers to Choosing Wisely in primary care: it’s not just about “the money.” J Gen Intern Med. 2017;32(2):140-142. doi:10.1007/s11606-016-3916-7 27822770 PMC5264685

[55] Schlesinger M, Grob R Treating, fast and slow: Americans’ understanding of and responses to low-value care. Milbank Q. 2017;95(1):70-116. doi:10.1111/1468-0009.12246 28266067 PMC5339390

[56] Schwartz AL, Chernew ME, Landon BE, McWilliams JM Changes in low-value services in year 1 of the Medicare Pioneer accountable care organization program. JAMA Intern Med. 2015;175(11):1815-1825. doi:10.1001/jamainternmed.2015.4525 26390323 PMC4928485

[57] Kullgren JT, Krupka E, Schachter A, Precommitting to choose wisely about low-value services: a stepped wedge cluster randomised trial. BMJ Qual Safe. 2018;27(5):355-364. doi:10.1136/bmjqs-2017-006699 29066616

[58] Ouayogodé MH, Meara E, Chang CH, Forgotten patients: ACO attribution omits those with low service use and the dying. Am J Manag Care. 2018;24(7):e207-e215. 30020755 PMC6089367

[59] Colla CH Swimming against the current—what might work to reduce low-value care? N Engl J Med. 2014;371(14):1280-1283. doi:10.1056/NEJMp1404503 25271601 PMC4499847

[60] Fendrick AM, Smith DG, Chernew ME Applying value-based insurance design to low-value health services. Health Aff (Millwood). 2010;29(11):2017-2021. doi:10.1377/hlthaff.2010.0878 21041741

[61] Henderson J, Bouck Z, Holleman R, Comparison of payment changes and choosing wisely recommendations for use of low-value laboratory tests in the United States and Canada. JAMA Intern Med. 2020;180(4):524-531. doi:10.1001/jamainternmed.2019.7143 32040158 PMC7042826

[62] Gruber J, Maclean JC, Wright B, Wilkinson E, Volpp KG The effect of increased cost-sharing on low-value service use. Health Econ. 2020;29(10):1180-1201. doi:10.1002/hec.4127 32686138

[63] Mafi JN, Godoy-Travieso P, Wei E, Evaluation of an intervention to reduce low-value preoperative care for patients undergoing cataract surgery at a safety-net health system. JAMA Intern Med. 2019;179(5):648-657. doi:10.1001/jamainternmed.2018.8358 30907922 PMC6503569

[64] Colla CH, Morden NE, Sequist TD, Mainor AJ, Li Z, Rosenthal MB Payer type and low-value care: comparing Choosing Wisely services across commercial and Medicare populations. Health Serv Res. 2018;53(2):730-746. doi:10.1111/1475-6773.12665 28217968 PMC5867100

[65] Lee VS, Kawamoto K, Hess R, Implementation of a value-driven outcomes program to identify high variability in clinical costs and outcomes and association with reduced cost and improved quality. JAMA. 2016;316(10):1061-1072. doi:10.1001/jama.2016.12226 27623461

[66] Angiolillo J, Rosenbloom ST, McPheeters M, Seibert Tregoning G, Rothman RL, Walsh CG Maintaining automated measurement of Choosing Wisely adherence across the *ICD 9* to *10* transition. J Biomed Inform. 2019;93:103142. doi:10.1016/j.jbi.2019.103142 30853653

