# Supplementary material for: Trends in Low-Value Health Service Use and Spending in the US Medicare Fee-for-Service Program, 2014-2018
Source: JAMA Netw Open. 2021 Feb 16;4(2):e2037328. doi: 10.1001/jamanetworkopen.2020.37328 (PMC7887655; doi:10.1001/jamanetworkopen.2020.37328)
Supplement: Supplement. — eAppendix. Sensitivity Analyses on the Impact of Demographic Trends eFigure. Study Design: 5 Repeated Cross-Sectional Cohorts, 2014-2018 eTable 1. Milliman MedInsight Health Waste Calculator Specifications for 32 Measures of Low-Value Care eTable 2. Three Sensitivity Analyses on Trends Including Measures With Relatively Little Reliance on Diagnosis Codes [file jamanetwopen-e2037328-s001.pdf]

Supplemental Online Content

Mafi JN, Reid RO, Baseman LH, et al. Trends in low-value health service use and spending in the US Medicare fee-for-service program, 2014-2018. *JAMA Netw Open*. 2021;4(2):e2037328. doi:10.1001/jamanetworkopen.2020.37328

eAppendix. Sensitivity Analyses on the Impact of Demographic Trends
eFigure. Study Design: 5 Repeated Cross-Sectional Cohorts, 2014-2018
eTable 1. Milliman MedInsight Health Waste Calculator Specifications for 32 Measures of Low-Value Care
eTable 2. Three Sensitivity Analyses on Trends Including Measures With Relatively Little Reliance on Diagnosis Codes

This supplemental material has been provided by the authors to give readers additional information about their work.

© 2021 Mafi JN et al. *JAMA Network Open.*

eAppendix. Sensitivity Analyses on the Impact of Demographic Trends

We performed sensitivity analyses assessing the potential impact of changing demographic trends. For the utilization analysis, we used a general sensitivity analysis method[1] in order to determine that to explain away the ratio that we see (36% of beneficiaries receiving any low-value service in 2014, and 33% of beneficiaries receiving any low-value service in 2018, for a risk ratio of 1.09), there would need to be a factor or factors that were both 40% more likely to occur in 2014 *and* 40% more likely to result in low-value services. Both of those possibilities seem unlikely.

For the spending analysis, using the demographic values in Table 1, it is not possible to construct a realistic scenario where the low-value spending for Black beneficiaries stays the same but the change in their proportion from 7.3% to 6.4% produces the decrease in spending from $52.70 to $46.90 per beneficiary (the only way to do so would be to suppose that the low-value spending among Black beneficiaries is $650.00 per beneficiary while it is $5.70 per beneficiary among all other race groups). The same is true for the other race category; the changes in demographics cannot explain the differences we see.

© 2021 Mafi JN et al. *JAMA Network Open.*

eFigure. Study Design: 5 Repeated Cross-Sectional Cohorts, 2014-2018

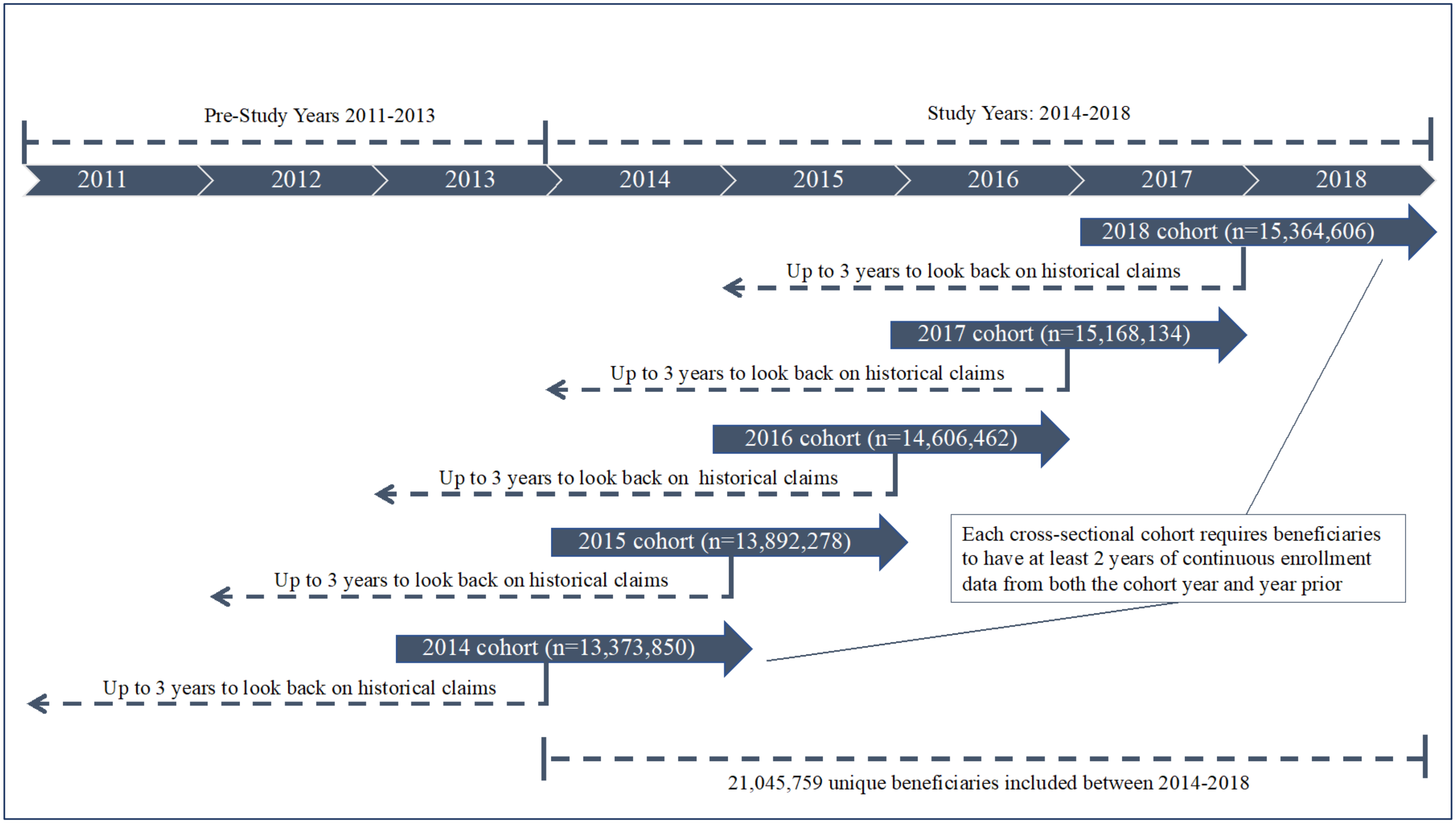

© 2021 Mafi JN et al. *JAMA Network Open.*

eTable 1. Milliman MedInsight Health Waste Calculator Specifications for 32 Measures of Low-Value Care

MEASURE ID & NAME	STARTING POPULATION	EXCLUSION	NOT WASTEFUL	LIKELY WASTEFUL	WASTEFUL	Last Updated	CITATIONS
AAPMR05 - Opioids for Acute Back Pain	All members aged 18 years and older with a prescription of opioids within 28 days of a diagnosis of low back pain.	None.	Members with a diagnosis of cancer or sickel cell anemia 180 days prior to the opioid prescription.	Members with a diagnosis of low back pain and a prescription for NSAIDs or tramadol or duloxetine within 90 days on or prior to the index event ((visit for a diagnosis of back pain).	All remaining.	Feb-18	http://annals.org/aim/fullarticle/2603228/noninvasive-treatments-acute-subacute-chronic-low-back-pain-clinical-practice

© 2021 Mafi JN et al. *JAMA Network Open.*

MEASURE ID & NAME	STARTING POPULATION	EXCLUSION	NOT WASTEFUL	LIKELY WASTEFUL	WASTEFUL	Last Updated	CITATIONS
ACC00 - Cardiac stress testing	All members aged 18 years and older with a service for cardiac stress testing.	Members from the starting population with:<br>· An inpatient admissions 30 days prior to the cardiac stress testing; or<br>- Emergency care or observation care within 1 day on or after the cardiac stress testing; or<br>· Coronary angiography on the day of the cardica stress tetsing; or<br>· PCI/CABG within 30 days on or after the cardiac stress testing.	Members with a service for:<br>- Stress testing (stress EKG, cardiac radionuclide imaging, and stress echo) and a diagnosis of cardiac symptoms or ventricular tachycardia within 90 days prior to the cardiac stress testing; or<br>· Stress CMR and a diagnosis of ventricular tachycardia within 90 days prior to the cardiac stress testing; or<br>· Advanced stress testing (cardiac radionuclide imaging, stress echo or stress CMR) and a diagnosis of cardiac conditions within 90 days prior to the cardiac stress testing; or<br>· Stress EKG with cardiac rehabilitation and a diagnosis of heart failure within 90 days prior to the cardiac stress testing; or<br>· Stress echocardiography and a diagnosis of valve disease or cardiomyopathy within 1 year prior to the cardiac stress testing; or<br>· Kidney or liver transplant and a diagnosis of pre-operative cardiovascular examination within 30 days prior to the pre-operative cardiac stress testing.	· Members with stress EKG and a diagnosis of cardiac conditions (heart failure, ventricular fibrillation, abnormal EKG findings, and coronary stenosis) within 90 days prior to the cardiac stress testing; or<br>· Member aged more than 40 years with 2 or more different risk factors (diabetes mellitus, hypertension, hyperlipidemia, obesity, coronary artery disease, peripheral artery disease) on the day of the stress testing.	All remaining.	Feb-18	http://annals.org/aim/article/1363528/screening-coronary-heart-disease-electrocardiography-u-s-preventive-services-task

© 2021 Mafi JN et al. *JAMA Network Open.*

MEASURE ID & NAME	STARTING POPULATION	EXCLUSION	NOT WASTEFUL	LIKELY WASTEFUL	WASTEFUL	Last Updated	CITATIONS
ACPY01 - Brain Imaging Studies (CT or MRI) for Simple Syncope	All members aged 18 years and older with a brain imaging study (CT or MRI) within 30 days of a diagnosis of syncope.	Members from the starting population with:<br>· An inpatient admission within the time period from the diagnosis of syncope to the brain imaging; or<br>· A competing diagnosis (CVA, intracranial hemorrhage, brain tumors etc.) within 90 days prior to the brain imaging; or<br>· Head Injury within 7 days prior to the brain imaging; or<br>· Diagnosis of benign or malignant tumors of the head and neck within 1 year prior to the brain imaging.	Members with a diagnosis of neurological deficits within the time period from the diagnosis of syncope to the brain imaging.	None.	All remaining.	Feb-18	http://www.acr.org/~/media/1C1F7C7A570D469A9C411D95067BDF94.pdf

© 2021 Mafi JN et al. *JAMA Network Open.*

MEASURE ID & NAME	STARTING POPULATION	EXCLUSION	NOT WASTEFUL	LIKELY WASTEFUL	WASTEFUL	Last Updated	CITATIONS
ACR01 - Imaging for Uncomplicated Headache	All members aged 18 years and older with a diagnosis of uncomplicated headache within 30 days prior to a head imaging.	Members from the starting population with:<br>- An inpatient admission within the time period from the diagnosis of headache to the head imaging; or<br>- Diagnosis of cancer or head trauma within 1 year prior to the head imaging; or<br>- Diagnosis of complicated sinusitis/mastoiditis/middle ear disease within 180 days prior to the head imaging.	All members with:<br>- Head MRI/ MRA AND Age >55 years AND raised ESR/temporal arteritis within the service unit; or<br>- CT/MRI/CT/MRA and diagnosis of complicated headache within the service unit; or<br>- Members with a diagnosis of underlying conditions (post traumatic headache, neurologic deficit, epilepsy, ataxia) who obtained a service for MRI/CT within the service unit; or<br>- MRI/CT and diagnosis of underlying conditions (Trigeminal headache, immunocompromised conditions) within the service unit; or<br>- MRI/CT and diagnosis of pregnancy without diagnosis of headache in the last 270 days prior to the index event; or<br>- MRI and diagnosis of meningitis/encephalitis or chronic conditions within the service unit; or<br>- MRI/MRA/CT and diagnosis of cerebrovascular event within the service unit.	All members with:<br>- CT/CTA and Age >55 years and raised ESR/temporal arteritis within the service unit; or<br>- CT/MRA/CTA and diagnosis of chronic conditions (Trigeminal headache immunocompromised conditions) within the service unit; or<br>- MRA/CTA and diagnosis of underlying conditions (post traumatic headache, neurologic deficit) within the service unit; or<br>- CT head and diagnosis of meningitis/encephalitis within the service unit; or<br>- Diagnosis of chronic headache within 1 year prior to the MRI head imaging.	All remaining	Feb-18	https://www.aafp.org/afp/2013/0515/p682.pdf

© 2021 Mafi JN et al. *JAMA Network Open.*

MEASURE ID & NAME	STARTING POPULATION	EXCLUSION	NOT WASTEFUL	LIKELY WASTEFUL	WASTEFUL	Last Updated	CITATIONS
AFP00 - Cervical Cancer Screening in Women	All female members with a cervical cancer screening testing service.	Members from the starting population with a diagnosis of HIV as far back in claims data prior to the cervical cancer screening testing.	· Members aged 21 years and older with a diagnosis of high risk conditions for developing cervical cancer or with gynecologic malignancy or dysplasia as far back in claims data; or<br>· Members aged 21 years or older and any documented abnormal Pap smear findings within 3 years prior to the cervical testing; or<br>· Members aged between 21 and 64 years who had cervical cytology once in 3 years and no codes of total hysterectomy as far back in claims data; or<br>· Members aged between 30 and 64 years who had cervical cytology and HPV testing on the same day and once in 5 years and had no codes of total hysterectomy as far back in claims data .	Members aged 21 years or older and a diagnosis of potential cervical cancer risk conditions (inflammatory disease of cervix uteri, co-infection with herpes simplex etc.) within 14 days prior to the cervical cancer screening testing.	All remaining.	Feb-18	http://www.uspreventiveservicestaskforce.org/Page/Document/RecommendationStatementFinal/cervical-cancer-screening

© 2021 Mafi JN et al. *JAMA Network Open.*

MEASURE ID & NAME	STARTING POPULATION	EXCLUSION	NOT WASTEFUL	LIKELY WASTEFUL	WASTEFUL	Last Updated	CITATIONS
AFP02 - Imaging for Low Back Pain	All members 18 years of age and older with a low back imaging service and diagnosis of low back pain within 6 weeks prior to the low back imaging.	Members from the starting population with a:<br>· Diagnosis of low back pain within 180 days prior to back imaging; or<br>· Lumbar spine surgery within 90 days prior to back Imaging; or<br>· Inpatient admission within 6 weeks prior to the back Imaging.	· Back MRI with a diagnosis of neurological deficits within the service unit; or<br>·MRI with diagnosis of other serious underlying conditions (cancer, immunosuppression) as far back prior to the back MRI; or<br>· Diagnosis of conditions requiring imaging (osteoporosis, trauma, drug abuse, infection) within 90 days of back MRI; or<br>· Age 70 years and older with back X-ray/CT lumbar spine without contrast/MRI; or<br>· X-ray/CT lumbar spine without contrast/MRI and long term steroid use; or<br>· Diagnosis of conditions requiring imaging (osteoporosis, trauma etc.) within 90 days of X-ray/CT lumbar spine without contrast.	Members with:<br>- Diagnosis of other serious underlying conditions (cancer, immunosuppression) and X-ray/CT lumbar spine as far back possible prior to the back imaging; or<br>· Diagnosis of any neurological deficits and a CT lumbar spine within the service unit.	All remaining.	Feb-18	https://acsearch.acr.org/docs/69483/Narrative/

© 2021 Mafi JN et al. *JAMA Network Open.*

MEASURE ID & NAME	STARTING POPULATION	EXCLUSION	NOT WASTEFUL	LIKELY WASTEFUL	WASTEFUL	Last Updated	CITATIONS
AFP03 - DEXA Screening for Osteoporosis	All women under 65 years of age and men 50-69 years of age who had a DEXA screeining with an office visit within 30 days prior to the DEXA screening.	Members from the starting population with a diagnosis of osteoporosis on the day of the DEXA or as far back in claims data prior to the DEXA screening.	Members with:<br>- A diagnosis of major risk factors for developing osteoporosis (vertebral compression fracture, malabsorption syndrome, osteopenia etc.) within 2 years prior to the DEXA screening; or<br>· At least two potential risk factors for developing osteoporosis (rheumatoid arthritis, hyperthyroidism etc.) within 2 years prior to the DEXA screening.	None.	All remaining.	Feb-18	http://annals.org/aim/article/746858/screening-osteoporosis-u-s-preventive-services-task-force-recommendation-statement

© 2021 Mafi JN et al. *JAMA Network Open.*

MEASURE ID & NAME	STARTING POPULATION	EXCLUSION	NOT WASTEFUL	LIKELY WASTEFUL	WASTEFUL	Last Updated	CITATIONS
AFP05 - Annual EKGs or Cardiac Screening in Asymptomatic Population	All members aged 18 years and older with a service for EKG or any other cardiac screening with an emergency/outpatient/inpatient visit within 14 days prior to the EKG or cardiac screening service.	Members from the starting population with:<br>- Diagnosis of inflammatory conditions such as arthritis, joint pains, myositis etc. within 14 days prior to the EKGs or other cardiac screening; or<br>- Low risk surgery within 30 days after the EKGs or other cardiac screening; or<br>- Inpatient stay on or 30 days prior to the EKGs or other cardiac screening.	Members with:<br>- Diagnosis of high risk markers for CHD (Diabetes, atherosclerotic disease etc.) within 2 years prior to the EKGs or other cardiac screening; or<br>- Diagnosis of two or more risk factors suggestive of intermediate CHD risk (Obesity, family history of ischemic heart disease etc.) within 2 years prior to the EKGs or other cardiac screening; or<br>- Diagnosis of two or more signs or symptoms suggestive of CHD (Chest pain, atrial flutter, tachycardia etc.) within 60 days on or prior to the EKGs or other cardiac screening.	None.	All remaining.	May-19	https://www.uspreventiveservicestaskforce.org/Page/Document/RecommendationStatementFinal/coronary-heart-disease-screening-with-electrocardiography

© 2021 Mafi JN et al. *JAMA Network Open.*

MEASURE ID & NAME	STARTING POPULATION	EXCLUSION	NOT WASTEFUL	LIKELY WASTEFUL	WASTEFUL	Last Updated	CITATIONS
AI02 - Immunoglobulin G (IgG) or Immunoglobulin E (IgE) Tests in the Evaluation of Allergy	All members with IgG or IgE testing and had a diagnosis of allergy within 30 days prior to the IgG or IgE testing.	None.	Members with IgE testing and:<br>- A diagnosis of eczema or dermatographism within 1 year prior to the IgE allergy test, or<br>· Children less than 15 years old.	· Members with a diagnosis of migraine and food allergy within 14 days prior to IgG allergy test; or<br>· Members with a diagnosis of atopic allergy within 14 days prior to the IgE allergy test.	All remaining.	Feb-18	http://www.aaaai.org/Aaaai/media/MediaLibrary/PDF%20Documents/Practice%20and%20Parameters/allergydiagnostictesting.pdf

© 2021 Mafi JN et al. *JAMA Network Open.*

MEASURE ID & NAME	STARTING POPULATION	EXCLUSION	NOT WASTEFUL	LIKELY WASTEFUL	WASTEFUL	Last Updated	CITATIONS
AI03 - Diagnostic Testing for Chronic Urticaria	All members with routine diagnostic testing and a diagnosis of chronic urticaria within 365 days prior to the diagnostic testing.	Members from the starting population with:<br>- A single allergy diagnostic testing service or where the count of the allergy test is more than one but without a diagnosis of urticaria.	None.	None.	All remaining.	Feb-18	https://www.aaaai.org/Aaaai/media/MediaLibrary/PDF%20Documents/Practice%20and%20Parameters/Urticaria-2014.pdf

© 2021 Mafi JN et al. *JAMA Network Open.*

MEASURE ID & NAME	STARTING POPULATION	EXCLUSION	NOT WASTEFUL	LIKELY WASTEFUL	WASTEFUL	Last Updated	CITATIONS
AN01 - Electroencephalography for Headaches	All members with an EEG and a diagnosis of headache within 30 days prior to the EEG.	Members from the starting population with:<br>- Inpatient admission in between the diagnosis of headache and a service for EEG.	Members with:<br>- Diagnosis of epilepsy or seizures within 180 days prior to EEG; or<br>- Diagnosis of abnormal involuntary movement between the diagnosis of headache and a service for EEG.	None.	All remaining.	Mar-16	http://staywell.com/wp-content/uploads/2013/12/Headache0113.pdf

© 2021 Mafi JN et al. *JAMA Network Open.*

MEASURE ID & NAME	STARTING POPULATION	EXCLUSION	NOT WASTEFUL	LIKELY WASTEFUL	WASTEFUL	Last Updated	CITATIONS
AN02 - Imaging of the Carotid Arteries for Simple Syncope	All members aged 18 years and older with a carotid duplex ultrasound imaging and a diagnosis of syncope within 14 days prior to the carotid artery duplex ultrasound.	Members from the starting population with:<br>- An inpatient admission between the diagnosis of simple syncope and a service of carotid duplex ultrasound.	Members with:<br>- Diagnosis of neurological deficit in between simple syncope and a service of carotid duplex ultrasound.	None.	All remaining.	Feb-18	https://www.ncbi.nlm.nih.gov/pmc/articles/PMC3295536/

© 2021 Mafi JN et al. *JAMA Network Open.*

MEASURE ID & NAME	STARTING POPULATION	EXCLUSION	NOT WASTEFUL	LIKELY WASTEFUL	WASTEFUL	Last Updated	CITATIONS
AO03 - Antibiotics Prescription for Adenoviral Conjunctivitis	All members with antibiotics prescription and a diagnosis of adenoviral conjunctivitis within 14 days prior to the antibiotics prescription.	None.	Members with:<br>- Diagnosis of secondary bacterial infection identified by associated otitis media or with symptoms of mucopurulent discharge from the eye within 14 days prior to the antibiotic prescription; or<br>- Diagnosis of skin conditions (such as impetigo, ecthyma, infected eczemas, cellulitis, erysipelas, infected wounds, MRSA infections etc.,) within 14 days prior to the antibiotic prescription.	None.	All remaining.	Feb-18	https://www.aoa.org/documents/CPG-11.pdf

© 2021 Mafi JN et al. *JAMA Network Open.*

MEASURE ID & NAME	STARTING POPULATION	EXCLUSION	NOT WASTEFUL	LIKELY WASTEFUL	WASTEFUL	Last Updated	CITATIONS
AOHN01 - CT Head/Brain for Sudden Onset Hearing Loss	All members with a CT scan of the head/brain within 7 days of sudden hearing loss.	Members from the starting population with:<br>- Competing diagnosis (meningitis, hemplagia, subarachnoid hemorrhage) within 30 days on or prior to CT of head or brain.	Members with:<br>- Diagnosis of acoustic neuroma/CVA within 90 days on or prior to the CT of head or brain and contraindications to MRI (pacemakers, metallic implants, severe claustrophobia, etc.) as far back as possible in member's history; or<br>- Diagnosis of pregnancy anytime between CT and diagnosis of sudden hearing loss; or<br>- Diagnosis of history of trauma involving the ear ear within 3 days prior to the CT of head or brain; or<br>- History of chronic ear disease or other related disease (Paget disease, fibrous dysplasia, encephalopathy or bone metastasis to the temporal bone, benign or malignant tumors of the petrous temporal bone, etc.) within 1 year prior to the CT of head or brain.	None.	All remaining.	Feb-18	http://journals.sagepub.com/doi/pdf/10.1177/0194599812436449

© 2021 Mafi JN et al. *JAMA Network Open.*

MEASURE ID & NAME	STARTING POPULATION	EXCLUSION	NOT WASTEFUL	LIKELY WASTEFUL	WASTEFUL	Last Updated	CITATIONS
AOHN04 - Imaging for Uncomplicated Acute Rhinosinusitis	All members with sinus imaging within 30 days of acute rhinosinusitis.	Members from the starting population with:<br>- Inpatient admissions within 30 days prior to imaging; or<br>- Competing diagnosis (headache, hearing loss, syncope and dizziness/vertigo) within 30 days prior to the imaging.	Members with:<br>- Diagnosis of chronic sinusitis within 30 days prior to imaging for sinus or head CT; or<br>- Diagnosis of acute or recurrent sinusitis on 3 different from dates within one year prior to imaging for sinus or head CT; or<br>- Diagnosis of complicated rhinosinusitis within 180 days prior to CT/MRI imaging; or<br>- Diagnosis of complicated rhinosinusitis (orbital or intracranial complications with ocular or neurologic deficits, preseptal or post septal cellulitis, sub periosteal abscess, orbital cellulitis or abscess) within 180 days prior to sinus CT/MRI.	Members with:<br>- Diagnosis of immunodeficiency or acute frontal or sphenoidal sinusitis within 180 days prior to sinus CT.	All remaining.	Feb-18	https://www.aafp.org/afp/2016/0715/p97.pdf

© 2021 Mafi JN et al. *JAMA Network Open.*

MEASURE ID & NAME	STARTING POPULATION	EXCLUSION	NOT WASTEFUL	LIKELY WASTEFUL	WASTEFUL	Last Updated	CITATIONS
AP00 - Antibiotics for acute upper respiratory and ear infections	All members aged 3 months and older with a prescription of oral antibiotics within 7 days of upper URI or ear infection (acute sinusitis, URI, viral respiratory illness or acute otitis externa).	Members from the starting population with:<br>- Diagnosis for comorbid conditions (HIV, malignant neoplasms or immunocompromised conditions etc.) within 1 year prior to the prescription of antibiotics; or<br>- Competing diagnosis (acute URI or acute external otitis in the presence of competing diagnosis (abscess, cellulitis, acute infections and other infectious conditions) within 30 days prior to the prescription of antibiotics; or<br>- Tympanostomy tube placement up to 2 years prior to prescription of antibiotics; or<br>- Diagnosis of otitis media within 14 days prior to the prescription of antibiotics.	Members with:<br>- Diagnosis for acute rhinosinusitis and sinusitis complications within 10 days prior to the antibiotic prescription; or<br>- Diagnosis of acute otitis externa and middle ear disease within 30 days prior to the antibiotic prescription; or<br>- Diagnosis for malignant otitis externa within 30 days prior to the antibiotic prescription.	None.	All remaining.	Jan-17	http://annals.org/aim/article/2481815/appropriate-antibiotic-use-acute-respiratory-tract-infection-adults-advice-high

© 2021 Mafi JN et al. *JAMA Network Open.*

MEASURE ID & NAME	STARTING POPULATION	EXCLUSION	NOT WASTEFUL	LIKELY WASTEFUL	WASTEFUL	Last Updated	CITATIONS
APA01 - Concurrent Use of Two or More Antipsychotic Medications	All members who were prescribed antipsychotics.	Members from the starting population with:<br>- Prescription for lithium within 60 days prior to antipsychotic prescription.	Members without any concurrent prescription of 2 different antipsychotic medication within 30 days consecutively for a period of 60 days prior antipsychotic prescription.	None.	All remaining.	Nov-19	http://www.choosingwisely.org/clinician-lists/american-psychiatric-association-routine-prescription-of-two-or-more-concurrent-antipsychotics/

© 2021 Mafi JN et al. *JAMA Network Open.*

MEASURE ID & NAME	STARTING POPULATION	EXCLUSION	NOT WASTEFUL	LIKELY WASTEFUL	WASTEFUL	Last Updated	CITATIONS
ASA01a - Preoperative Baseline Laboratory Studies	All members aged 2 years or older with a baseline laboratory testing within 30 days prior to a low risk non-cardiac surgery.	Members from the starting population with:<br>- E&M visit for emergency care, observation or urgent care within one day prior to the Pre-operative testing; or<br>- Electrolyte testing laboratory related services and prescription of medications such as digoxin, diuretics and angiotensin converting enzyme inhibitors or angiotensin receptor blockers within 90 days prior to the prior Pre-operative testing; or<br>- Diagnosis of endocrine, liver or renal disorders within 180 days prior to the prior Pre-operative testing; or<br>- Diagnosis of history of anemia or history suggestive of recent blood loss within 180 days prior to the Pre-operative CBC testing; or<br>- Coagulation testing related services and a diagnosis of coagulation disorders within 180 days prior to the Pre-operative CBC testing; or<br>- Prescription of anticoagulant medications within 90 days on or prior to coagulation testing related services.	Members with:<br>- Urinalysis prior to urologic procedure or after the diagnosis of urinary symptoms or disorders.	None.	All remaining.	May-19	http://anesthesiology.pubs.asahq.org/article.aspx?articleid=2443414

© 2021 Mafi JN et al. *JAMA Network Open.*

MEASURE ID & NAME	STARTING POPULATION	EXCLUSION	NOT WASTEFUL	LIKELY WASTEFUL	WASTEFUL	Last Updated	CITATIONS
ASA01b - Preoperative EKG, Chest X-Ray and Pulmonary Function Testing	All members aged 2 years or older with an EKG, chest X-ray and pulmonary function within 30 days prior to low risk surgery.	Members from the starting population with:<br>- E&M visit for emergency care, observation or urgent care on or within one day prior to the Pre-operative testing.	Members with:<br>- Diagnosis of cardiovascular risk factors within 90 days prior to the Pre-operative testing; or<br>-Signs and symptoms of cardiovascular and cardiopulmonary disease disease within 30 days prior to the Pre-operative testing; or<br>- Diagnosis of underlying pulmonary disease within 90 days prior to the Pre-operative testing.	None.	All remaining.	May-19	http://anesthesiology.pubs.asahq.org/article.aspx?articleid=2443414

© 2021 Mafi JN et al. *JAMA Network Open.*

MEASURE ID & NAME	STARTING POPULATION	EXCLUSION	NOT WASTEFUL	LIKELY WASTEFUL	WASTEFUL	Last Updated	CITATIONS
ASA02 - Preoperative Cardiac Echocardiography or Stress Testing	All members aged 18 years or older with an echocardiography or stress testing within 30 days prior to a low or intermediate risk non-cardiac surgery.	Members from the starting population with:<br>- Inpatient admission on or 30 days prior to the Pre-operative echocardiography or stress testing; or<br>- E&M visit for emergency care, observation or urgent care on or within one day prior to Pre-operative echocardiography or stress testing; or<br>- Diagnosis of high risk markers for CHD within 2 years prior to Pre-operative echocardiography or stress testing.	Members with:<br>- Diagnosis of two or more different signs or symptoms suggestive of CHD within 60 days prior to Pre-operative echocardiography or stress testing.	None.	All remaining.	May-19	http://circ.ahajournals.org/content/130/24/2215.long

© 2021 Mafi JN et al. *JAMA Network Open.*

MEASURE ID & NAME	STARTING POPULATION	EXCLUSION	NOT WASTEFUL	LIKELY WASTEFUL	WASTEFUL	Last Updated	CITATIONS
DOR124 - Renal Artery Revascularization	All members with renal artery revascularization.	None.	Members with:<br>- Diagnosis of fibromuscular dysplasia within 90 days prior to renal artery revascularization.	Members with:<br>- Diagnosis of malignant hypertension within 90 days prior to renal artery revascularization; or<br>- Diagnosis of chronic kidney disease stage III to stage VI within 90 days prior to renal artery revascularization; or<br>- Diagnosis of pulmonary edema or acute coronary syndrome within 90 days prior to renal artery revascularization.	All remaining.	Feb-18	http://www.nejm.org/doi/full/10.1056/NEJMoa0905368

© 2021 Mafi JN et al. *JAMA Network Open.*

MEASURE ID & NAME	STARTING POPULATION	EXCLUSION	NOT WASTEFUL	LIKELY WASTEFUL	WASTEFUL	Last Updated	CITATIONS
DOR21 - Arthroscopic Lavage and Debridement for Knee Osteoarthritis	All members aged 18 years and older with arthroscopic lavage or debridement within 365 days of knee osteoarthritis.	None.	None.	None.	All remaining.	Feb-18	http://www.aaos.org/research/guidelines/TreatmentofOsteoarthritisoftheKneeGuideline.pdf

© 2021 Mafi JN et al. *JAMA Network Open.*

MEASURE ID & NAME	STARTING POPULATION	EXCLUSION	NOT WASTEFUL	LIKELY WASTEFUL	WASTEFUL	Last Updated	CITATIONS
DOR85 - Antidepressants Monotherapy in Bipolar Disorder	All members with a prescription of antidepressants within 3 days of bipolar disorder.	None.	Members with:<br>- Prescription for mood stabilizers within 90 days prior to antidepressants monotherapy.	None.	All remaining.	Feb-18	https://www.healthquality.va.gov/bipolar/bd_306_sum.pdf

© 2021 Mafi JN et al. *JAMA Network Open.*

MEASURE ID & NAME	STARTING POPULATION	EXCLUSION	NOT WASTEFUL	LIKELY WASTEFUL	WASTEFUL	Last Updated	CITATIONS
GE01 - Colorectal Cancer Screening in Adults 50 Years and Older	All members aged 50 years and older with colorectal cancer screening.	Members from the starting population with:<br>- Diagnosis of colorectal cancer, family or personal history of colorectal cancer or colon adenoma as far back as possible in members' history; or<br>- Service for total colectomy as far back as possible in members' history.	Members with:<br>- FOBT once in a year; or<br>- Immunochemical-based fecal occult blood testing once in a year; or<br>- FIT-DNA once in a year; or<br>- Flexible sigmoidoscopy once in 5 years; or<br>- CT colonography once in 5 years; or<br>- Colonoscopy once in 10 years.	None.	All remaining.	Feb-18	http://www.cancer.org/acs/groups/cid/documents/webcontent/003170-pdf.pdf

© 2021 Mafi JN et al. *JAMA Network Open.*

MEASURE ID & NAME	STARTING POPULATION	EXCLUSION	NOT WASTEFUL	LIKELY WASTEFUL	WASTEFUL	Last Updated	CITATIONS
JH001 - CT Scans for Emergency Room Evaluation of Dizziness	All members aged 18 years and older with CT scan within 1 day of dizziness.	Members from the starting population with:<br>- Diagnosis of Comorbid conditions (headache or hearing loss or complicated sinusitis/mastoiditis/middle ear disorder) within 30 days prior to CT scan; or<br>- Inpatient admission within the service unit.	Members with:<br>- Competing diagnosis (a history of recent head injury or other brain conditions) within 7 days prior to CT scan; or<br>- Diagnosis of benign or malignant tumors of the head and neck within one year prior to CT scan.	None.	All remaining.	Feb-18	https://www.ncbi.nlm.nih.gov/pmc/articles/PMC2676794/pdf/nihms102245.pdf

© 2021 Mafi JN et al. *JAMA Network Open.*

MEASURE ID & NAME	STARTING POPULATION	EXCLUSION	NOT WASTEFUL	LIKELY WASTEFUL	WASTEFUL	Last Updated	CITATIONS
SCCT01 - Coronary Artery Calcium Scoring for Known CAD	All members aged 18 years and older with calcium scoring within 1 year of coronary artery disease.	None.	None.	None.	All starting population.	Feb-18	http://circ.ahajournals.org/content/129/25_suppl_2/S49.long

© 2021 Mafi JN et al. *JAMA Network Open.*

MEASURE ID & NAME	STARTING POPULATION	EXCLUSION	NOT WASTEFUL	LIKELY WASTEFUL	WASTEFUL	Last Updated	CITATIONS
SCP01 - Screening for Vitamin D Deficiency	All members with vitamin-D testing (25-OH-Vitamin D and 1, 25-dihydroxyvitamin D testing).	None.	Members with:<br>- Diagnosis of chronic conditions within one year prior to 25-OH-vitamin D testing; or<br>- Diagnosis of risk factors within 90 days prior to 25-OH-vitamin D testing; or<br>- Prescription for high risk medications within 90 days prior to 25-OH-vitamin D testing; or<br>- Diagnosis of pregnancy and obesity on the day of 25-OH-vitamin D testing; or<br>- Age 65 years or older with any history of falls or a history of non-traumatic fractures within 1 year prior to 25-OH-vitamin D testing; or<br>- Diagnosis of inherited or acquired disorders of vitamin D and phosphate metabolism within 90 days prior to 1,25 (OH)2 -vitamin D testing.	None.	All remaining.	Feb-18	http://annals.org/aim/fullarticle/1938935/screening-vitamin-d-deficiency-adults-u-s-preventive-services-task

© 2021 Mafi JN et al. *JAMA Network Open.*

MEASURE ID & NAME	STARTING POPULATION	EXCLUSION	NOT WASTEFUL	LIKELY WASTEFUL	WASTEFUL	Last Updated	CITATIONS
SCP05 - Bleeding Time Testing	All members with a bleeding test.	None.	None.	None.	All starting population.	Feb-18	http://www.ajmc.com/journals/issue/2010/2010-09-vol16-n09/ajmc_10sep_wu_xcl_e220to227

© 2021 Mafi JN et al. *JAMA Network Open.*

MEASURE ID & NAME	STARTING POPULATION	EXCLUSION	NOT WASTEFUL	LIKELY WASTEFUL	WASTEFUL	Last Updated	CITATIONS
SNP01 - Peripherally Inserted Central Catheters in Stage III-V CKD Patients	All members with PICC placement within 1 year of stage III-V Chronic Kidney disease (III-V).	None.	Members with:<br>- Nephrology consult within 7 days prior to the PICC line insertion.	None.	All remaining.	May-19	http://c.ymcdn.com/sites/www.asdin.org/resource/resmgr/imported/ASDINVeinPreservation.pdf

© 2021 Mafi JN et al. *JAMA Network Open.*

MEASURE ID & NAME	STARTING POPULATION	EXCLUSION	NOT WASTEFUL	LIKELY WASTEFUL	WASTEFUL	Last Updated	CITATIONS
SNP04 - NSAIDs for Hypertension, Heart failure or Chronic Kidney Disease	All members aged 18 years and older with a prescription of NSAIDS with a diagnosis of hypertension, heart failure, or chronic kidney disease within 7 days prior to the prescription.	None.	Members with:<br>- Prescription of aspirin or prescription of topical NSAIDs.	None.	All remaining.	Feb-18	http://circ.ahajournals.org/content/123/21/2434.long

© 2021 Mafi JN et al. *JAMA Network Open.*

MEASURE ID & NAME	STARTING POPULATION	EXCLUSION	NOT WASTEFUL	LIKELY WASTEFUL	WASTEFUL	Last Updated	CITATIONS
SNUC01 - Coronary Angiography	All members aged 18 years or older with coronary angiography.	Members from the starting population with:<br>- Cardiac transplant status or congenital cardiac anomalies as far back as possible in members' history.	Members with:<br>- Cardiac conditions (acute coronary syndrome, myocardial infarction, heart failure, or ventricular fibrillation or ventricular tachycardia) within 30 days prior to coronary angiography; or<br>- A service for cardiac valve surgeries within 30 days prior to coronary angiography<br>- Diagnosis of Obstructive coronary artery disease or PCI/CABG as far back with symptoms of chronic heart disease or abnormal cardiovascular study results within 30 days prior to coronary angiography; or<br>- A service for stress test and symptoms of chronic heart disease and abnormal cardiovascular study results within 14 days prior to coronary angiography.	Members with:<br>- Diagnosis of other cardiac conditions (atrial fibrillation, heart block, left bundle branch block, valve disease, cardiomyopathies, pericardial disease and stenosis/ regurgitation) on the day of Coronary angiography; or<br>-Diagnosis of pre-operative cardiovascular examination and 3 or more different high risk conditions (ischemic heart disease, diabetes mellitus, renal insufficiency, CVA on the day of coronary angiography; or<br>- A service for stress test with symptoms of chronic heart disease or abnormal cardiovascular study results within 14 days prior to coronary angiography.	All remaining.	Feb-18	http://www.sciencedirect.com/science/article/pii/S0735109713061470?via%3Dihub

© 2021 Mafi JN et al. *JAMA Network Open.*

MEASURE ID & NAME	STARTING POPULATION	EXCLUSION	NOT WASTEFUL	LIKELY WASTEFUL	WASTEFUL	Last Updated	CITATIONS
STHS05 - Pulmonary Function Testing Before Cardiac Surgery	All members aged 18 years and older with pulmonary function testing within 30 days prior to cardiac surgery.	None.	Members with a diagnosis of:<br>- Any underlying pulmonary disease within 90 days prior to pulmonary function testing; or<br>- Respiratory symptoms within 30 days prior to pulmonary function testing.	None.	All remaining.	Feb-18	https://jamanetwork.com/journals/jama/fullarticle/2510916

© 2021 Mafi JN et al. *JAMA Network Open.*

eTable 2. Three Sensitivity Analyses on Trends Including Measures With Relatively Little Reliance on Diagnosis Codes

Utilization trends per 1,000 Medicare Beneficiaries among 13 measures with no ICD9 or ICD10 codes used to classify "not wasteful" services or use at least 8 ICD9 codes for every 10 ICD 10 codes used to classify "not wasteful" services																				
Measure	2014 Services	std_err_services_2014	CI 2014		2015 Services	std_err_services_2015	CI 2015		2016 Services	std_err_services_2016	CI 2016		2017 Services	std_err_services_2017	CI 2017		2018 Services	std_err_services_2018	CI 2018	
AI02-Immunoglobulin G (IgG) or Immunoglobulin E (IgE) Tests in the Evaluation of Allergy	0.46	0.00	0.45	0.47	0.51	0.01	0.50	0.52	0.71	0.01	0.70	0.72	0.80	0.01	0.78	0.82	0.84	0.01	0.82	0.86
AI03-Diagnostic Testing for Chronic Urticaria	0.09	0.00	0.09	0.09	0.09	0.00	0.09	0.09	0.10	0.00	0.10	0.10	0.11	0.00	0.11	0.11	0.11	0.00	0.11	0.11
APA01-Concurrent Use of Two or More Antipsychotic Medications	32.56	0.70	31.19	33.93	31.09	0.66	29.79	32.39	29.55	0.63	28.32	30.78	29.31	0.63	28.08	30.54	28.15	0.02	28.11	28.19
ASA02-Preoperative Cardiac Echocardiography or Stress Testing	0.15	0.00	0.15	0.15	0.13	0.00	0.13	0.13	0.14	0.00	0.14	0.14	0.14	0.00	0.14	0.14	0.13	0.01	0.12	0.14
DOR21-Arthroscopic Lavage and Debridement for Knee Osteoarthritis	0.11	0.00	0.11	0.11	0.11	0.00	0.11	0.11	0.11	0.00	0.11	0.11	0.10	0.00	0.10	0.10	0.09	0.00	0.08	0.10
DOR85-Antidepressants Monotherapy in Bipolar Disorder	0.90	0.01	0.87	0.93	0.96	0.02	0.93	0.99	0.89	0.02	0.86	0.92	0.93	0.02	0.89	0.97	0.95	0.02	0.92	0.98
GE01-Colorectal Cancer Screening in Adults 50 Years and Older	35.22	0.03	35.17	35.27	33.19	0.02	33.15	33.23	31.99	0.02	31.95	32.03	31.61	0.02	31.57	31.65	30.43	0.00	30.43	30.43
SCCT01-Coronary Artery Calcium Scoring for Known CAD	0.09	0.00	0.09	0.09	0.12	0.00	0.12	0.12	0.15	0.00	0.15	0.15	0.20	0.00	0.20	0.20	0.22	0.00	0.22	0.22
SCP05-Bleeding Time Testing	0.26	0.00	0.25	0.27	0.21	0.01	0.20	0.22	0.12	0.00	0.11	0.13	0.09	0.00	0.09	0.09	0.07	0.03	0.02	0.12
SNP01-Peripherally Inserted Central Catheters in Stage III-V CKD Patients	0.74	0.00	0.73	0.75	0.79	0.00	0.78	0.80	0.86	0.00	0.85	0.87	0.89	0.00	0.88	0.90	0.93	0.00	0.92	0.94
SNP04-NSAIDs for Hypertension, Heart failure or Chronic Kidney Disease	58.39	0.09	58.21	58.57	60.77	0.09	60.60	60.94	61.52	0.08	61.35	61.69	60.32	0.08	60.17	60.47	56.94	0.00	56.94	56.94
SNUC01-Coronary Angiography	1.41	0.00	1.40	1.42	1.40	0.00	1.39	1.41	1.33	0.00	1.32	1.34	1.38	0.00	1.37	1.39	1.39	0.00	1.38	1.40
STHS05-Pulmonary Function Testing Before Cardiac Surgery	0.08	0.00	0.08	0.08	0.09	0.00	0.09	0.09	0.10	0.00	0.10	0.10	0.13	0.00	0.13	0.13	0.12	0.00	0.11	0.13
Total	130.46	0.70	129.08	131.84	129.46	0.67	128.15	130.77	127.57	0.64	126.33	128.81	126.01	0.63	124.77	127.25	120.37	0.04	120.29	120.45

© 2021 Mafi JN et al. *JAMA Network Open.*

Claim-level spending trends per 1,000 Medicare Beneficiaries among 13 measures with no ICD9 or ICD10 codes used to classify "not wasteful" services or use at least 8 ICD9 codes for every 10 ICD 10 codes used to classify "not wasteful" services																				
Measure	2014 Claim Spend	std_err_claimspend_2014	CI 2014		2015 Claim Spend	std_err_claimspend_2015	CI 2015		2016 Claim Spend	std_err_claimspend_2016	CI 2016		2017 Claim Spend	std_err_claimspend_2017	CI 2017		2018 Claim Spend	std_err_claimspend_2018	CI 2018	
AI02-Immunoglobulin G (IgG) or Immunoglobulin E (IgE) Tests in the Evaluation of Allergy	103.5	6.0	91.7	115.2	106.1	7.4	91.6	120.7	150.5	15.7	119.8	181.3	165.8	7.9	150.4	181.2	160.2	8.1	144.2	176.1
AI03-Diagnostic Testing for Chronic Urticaria	29.9	3.3	23.5	36.4	36.1	5.6	25.1	47.0	35.8	3.7	28.4	43.1	47.8	4.3	39.4	56.3	44.4	43.6	-41.1	129.9
APA01-Concurrent Use of Two or More Antipsychotic Medications	7816.9	328.5	7173.0	8460.8	10671.3	507.7	9676.1	11666.4	8971.6	476.9	8036.9	9906.3	9548.7	521.4	8526.7	10570.7	9912.0	20.3	9872.1	9951.9
ASA02-Preoperative Cardiac Echocardiography or Stress Testing	114.3	5.1	104.3	124.4	89.6	2.9	83.9	95.3	92.6	4.8	83.2	102.1	87.5	2.5	82.6	92.4	85.0	23.3	39.4	130.7
DOR21-Arthroscopic Lavage and Debridement for Knee Osteoarthritis	149.6	3.4	143.0	156.2	152.4	3.1	146.3	158.6	146.2	3.3	139.7	152.7	126.8	2.8	121.3	132.4	124.7	33.6	58.8	190.6
DOR85-Antidepressants Monotherapy in Bipolar Disorder	58.0	2.8	52.4	63.5	52.2	4.3	43.8	60.6	50.5	4.0	42.5	58.4	52.5	5.2	42.2	62.8	50.0	3.3	43.4	56.6
GE01-Colorectal Cancer Screening in Adults 50 Years and Older	11057.0	70.2	10919.5	11194.5	10634.5	47.4	10541.5	10727.4	9995.9	34.8	9927.8	10064.1	9589.2	40.0	9510.8	9667.6	9593.0	3.0	9587.2	9598.8
SCCT01-Coronary Artery Calcium Scoring for Known CAD	38.9	2.5	34.1	43.7	43.1	2.2	38.7	47.4	44.3	2.1	40.3	48.4	62.1	2.1	57.9	66.2	72.7	11.8	49.6	95.7
SCP05-Bleeding Time Testing	318.9	17.3	285.0	352.8	251.1	16.3	219.1	283.0	124.1	8.5	107.4	140.8	121.3	11.1	99.6	143.1	107.2	106.6	-101.9	316.2
SNP01-Peripherally Inserted Central Catheters in Stage III-V CKD Patients	2692.5	74.4	2546.7	2838.3	5482.4	1937.4	1685.1	9279.8	5501.1	122.2	5261.6	5740.6	5379.4	121.6	5141.0	5617.7	5797.8	151.8	5500.2	6095.4
SNP04-NSAIDs for Hypertension, Heart failure or Chronic Kidney Disease	4652.3	29.9	4593.6	4710.9	3058.7	26.1	3007.5	3109.9	2216.5	18.4	2180.4	2252.5	1922.1	17.9	1887.1	1957.2	1703.8	0.3	1703.2	1704.4
SNUC01-Coronary Angiography	5560.3	44.9	5472.3	5648.4	5366.9	41.2	5286.2	5447.7	4893.3	35.8	4823.1	4963.6	5185.1	35.9	5114.7	5255.5	5398.7	27.1	5345.5	5451.9
STHS05-Pulmonary Function Testing Before Cardiac Surgery	58.5	3.5	51.7	65.4	62.2	3.9	54.6	69.7	72.9	4.2	64.8	81.1	92.6	4.9	83.0	102.3	86.4	34.7	18.3	154.5
Total	32650.5	348.9	31966.8	33334.3	36006.5	2004.1	32078.4	39934.5	32295.4	495.6	31324.1	33266.7	32381.0	538.7	31325.2	33436.8	33135.9	201.4	32741	33530.7

© 2021 Mafi JN et al. *JAMA Network Open.*

Line-level spending trends per 1,000 Medicare Beneficiaries among 13 measures with no ICD9 or ICD10 codes used to classify "not wasteful" services or use at least 8 ICD9 codes for every 10 ICD 10 codes used to classify "not wasteful" services

Measure	2014 Line Spend	std_err_linespend_2014	CI 2014		2015 Line Spend	std_err_linespend_2015	CI 2015		2016 Line Spend	std_err_linespend_2016	CI 2016		2017 Line Spend	std_err_linespend_2017	CI 2017		2018 Line Spend	std_err_linespend_2018	CI 2018	
AI02-Immunoglobulin G (IgG) or Immunoglobulin E (IgE) Tests in the Evaluation of Allergy	36.5	0.8	35.0	38.0	39.1	0.9	37.4	40.7	53.7	1.0	51.7	55.7	56.6	0.9	54.9	58.3	54.6	0.9	52.9	56.3
AI03-Diagnostic Testing for Chronic Urticaria	7.9	0.5	7.0	8.9	7.2	0.4	6.4	8.1	10.4	0.6	9.3	11.6	8.5	0.4	7.7	9.4	7.2	3.0	1.2	13.1
APA01-Concurrent Use of Two or More Antipsychotic Medications	7816.6	328.5	7172.7	8460.5	7251.8	339.0	6587.4	7916.2	5975.4	315.0	5358.0	6592.7	6338.2	340.4	5671.0	7005.5	6555.4	13.1	6529.7	6581.0
ASA02-Preoperative Cardiac Echocardiography or Stress Testing	79.5	1.7	76.2	82.8	67.6	1.4	64.8	70.3	65.0	1.2	62.7	67.3	64.9	1.2	62.6	67.2	62.6	9.3	44.3	80.8
DOR21-Arthroscopic Lavage and Debridement for Knee Osteoarthritis	149.6	3.4	143.0	156.2	152.4	3.1	146.3	158.6	146.2	3.3	139.7	152.7	126.8	2.8	121.3	132.4	124.7	33.6	58.8	190.6
DOR85-Antidepressants Monotherapy in Bipolar Disorder	58.0	2.8	52.4	63.5	52.2	4.3	43.8	60.6	50.5	4.0	42.5	58.4	52.5	5.2	42.2	62.8	50.0	3.3	43.4	56.6
GE01-Colorectal Cancer Screening in Adults 50 Years and Older	6085.0	19.8	6046.2	6123.8	5968.7	18.9	5931.6	6005.7	5748.5	17.7	5713.9	5783.1	5347.1	17.2	5313.5	5380.8	5524.8	0.8	5523.3	5526.4
SCCT01-Coronary Artery Calcium Scoring for Known CAD	33.3	2.4	28.5	38.0	36.2	2.2	31.9	40.5	36.3	2.0	32.3	40.3	51.3	2.1	47.2	55.4	60.8	11.6	38.0	83.6
SCP05-Bleeding Time Testing	1.6	0.1	1.4	1.7	1.1	0.1	1.0	1.3	0.7	0.0	0.7	0.8	0.6	0.0	0.5	0.6	0.4	0.3	-0.3	1.0
SNP01-Peripherally Inserted Central Catheters in Stage III-V CKD Patients	1610.2	69.0	1475.0	1745.4	2252.4	117.1	2022.9	2481.9	3940.8	111.3	3722.6	4159.1	3626.2	106.9	3416.7	3835.7	3596.0	122.4	3356.2	3835.9
SNP04-NSAIDs for Hypertension, Heart failure or Chronic Kidney Disease	4652.2	29.9	4593.6	4710.8	3058.7	26.1	3007.5	3109.9	2216.5	18.4	2180.4	2252.5	1922.2	17.9	1887.1	1957.2	1703.8	0.3	1703.3	1704.4
SNUC01-Coronary Angiography	3467.7	20.8	3426.9	3508.5	3118.0	17.7	3083.3	3152.7	2952.9	17.5	2918.5	2987.2	3301.6	19.7	3263.0	3340.3	3374.5	14.4	3346.3	3402.7
STHS05-Pulmonary Function Testing Before Cardiac Surgery	3.8	0.1	3.5	4.0	1.3	0.1	1.2	1.5	1.3	0.1	1.2	1.5	1.8	0.1	1.6	2.0	1.8	0.8	0.3	3.3
Total	24001.8	338.3	23338.8	24664.9	22006.6	360.6	21300.0	22713.3	21198.3	335.5	20540.6	21855.9	20898.4	358.3	20196.2	21600.6	21116.6	129.3	20863.1	21370.1

© 2021 Mafi JN et al. *JAMA Network Open.*

Utilization trends per 1,000 Medicare Beneficiaries among 10 measures with no ICD9 or ICD10 codes used to classify "not wasteful" services

Measure	2014 Services	std_err_services_2014	CI 2014		2015 Services	std_err_services_2015	CI 2015		2016 Services	std_err_services_2016	CI 2016		2017 Services	std_err_services_2017	CI 2017		2018 Services	std_err_services_2018	CI 2018	
AI03-Diagnostic Testing for Chronic Urticaria	0.09	0.00	0.09	0.09	0.09	0.00	0.09	0.09	0.10	0.00	0.10	0.10	0.11	0.00	0.11	0.11	0.11	0.00	0.11	0.11
APA01-Concurrent Use of Two or More Antipsychotic Medications	32.56	0.70	31.19	33.93	31.09	0.66	29.79	32.39	29.55	0.63	28.32	30.78	29.31	0.63	28.08	30.54	28.15	0.02	28.11	28.19
ASA02-Preoperative Cardiac Echocardiography or Stress Testing	0.15	0.00	0.15	0.15	0.13	0.00	0.13	0.13	0.14	0.00	0.14	0.14	0.14	0.00	0.14	0.14	0.13	0.01	0.12	0.14
DOR21-Arthroscopic Lavage and Debridement for Knee Osteoarthritis	0.11	0.00	0.11	0.11	0.11	0.00	0.11	0.11	0.11	0.00	0.11	0.11	0.10	0.00	0.10	0.10	0.09	0.00	0.08	0.10
DOR85-Antidepressants Monotherapy in Bipolar Disorder	0.90	0.01	0.87	0.93	0.96	0.02	0.93	0.99	0.89	0.02	0.86	0.92	0.93	0.02	0.89	0.97	0.95	0.02	0.92	0.98
GE01-Colorectal Cancer Screening in Adults 50 Years and Older	35.22	0.03	35.17	35.27	33.19	0.02	33.15	33.23	31.99	0.02	31.95	32.03	31.61	0.02	31.57	31.65	30.43	0.00	30.43	30.43
SCCT01-Coronary Artery Calcium Scoring for Known CAD	0.09	0.00	0.09	0.09	0.12	0.00	0.12	0.12	0.15	0.00	0.15	0.15	0.20	0.00	0.20	0.20	0.22	0.00	0.22	0.22
SCP05-Bleeding Time Testing	0.26	0.00	0.25	0.27	0.21	0.01	0.20	0.22	0.12	0.00	0.11	0.13	0.09	0.00	0.09	0.09	0.07	0.03	0.02	0.12
SNP01-Peripherally Inserted Central Catheters in Stage III-V CKD Patients	0.74	0.00	0.73	0.75	0.79	0.00	0.78	0.80	0.86	0.00	0.85	0.87	0.89	0.00	0.88	0.90	0.93	0.00	0.92	0.94
SNP04-NSAIDs for Hypertension, Heart failure or Chronic Kidney Disease	58.39	0.09	58.21	58.57	60.77	0.09	60.60	60.94	61.52	0.08	61.35	61.69	60.32	0.08	60.17	60.47	56.94	0.00	56.94	56.94
Total	128.51	0.70	127.13	129.89	127.46	0.67	126.15	128.77	125.43	0.64	124.19	126.67	123.70	0.63	122.46	124.94	118.02	0.04	117.94	118.10

© 2021 Mafi JN et al. *JAMA Network Open.*

Measure	2014 Claim Spend	std_err_claimspend_2014	CI 2014		2015 Claim Spend	std_err_claimspend_2015	CI 2015		2016 Claim Spend	std_err_claimspend_2016	CI 2016		2017 Claim Spend	std_err_claimspend_2017	CI 2017		2018 Claim Spend	std_err_claimspend_2018	CI 2018	
Claim-level spending trends per 1,000 Medicare Beneficiaries among 10 measures with no ICD9 or ICD10 codes used to classify "not wasteful" services																				
AI03-Diagnostic Testing for Chronic Urticaria	29.9	3.3	23.5	36.4	36.1	5.6	25.1	47.0	35.8	3.7	28.4	43.1	47.8	4.3	39.4	56.3	44.4	43.6	-41.1	129.9
APA01-Concurrent Use of Two or More Antipsychotic Medications	7816.9	328.5	7173.0	8460.8	10671.3	507.7	9676.1	11666.4	8971.6	476.9	8036.9	9906.3	9548.7	521.4	8526.7	10570.7	9912.0	20.3	9872.1	9951.9
ASA02-Preoperative Cardiac Echocardiography or Stress Testing	114.3	5.1	104.3	124.4	89.6	2.9	83.9	95.3	92.6	4.8	83.2	102.1	87.5	2.5	82.6	92.4	85.0	23.3	39.4	130.7
DOR21-Arthroscopic Lavage and Debridement for Knee Osteoarthritis	149.6	3.4	143.0	156.2	152.4	3.1	146.3	158.6	146.2	3.3	139.7	152.7	126.8	2.8	121.3	132.4	124.7	33.6	58.8	190.6
DOR85-Antidepressants Monotherapy in Bipolar Disorder	58.0	2.8	52.4	63.5	52.2	4.3	43.8	60.6	50.5	4.0	42.5	58.4	52.5	5.2	42.2	62.8	50.0	3.3	43.4	56.6
GE01-Colorectal Cancer Screening in Adults 50 Years and Older	11057.0	70.2	10919.5	11194.5	10634.5	47.4	10541.5	10727.4	9995.9	34.8	9927.8	10064.1	9589.2	40.0	9510.8	9667.6	9593.0	3.0	9587.2	9598.8
SCCT01-Coronary Artery Calcium Scoring for Known CAD	38.9	2.5	34.1	43.7	43.1	2.2	38.7	47.4	44.3	2.1	40.3	48.4	62.1	2.1	57.9	66.2	72.7	11.8	49.6	95.7
SCP05-Bleeding Time Testing	318.9	17.3	285.0	352.8	251.1	16.3	219.1	283.0	124.1	8.5	107.4	140.8	121.3	11.1	99.6	143.1	107.2	106.6	-101.9	316.2
SNP01-Peripherally Inserted Central Catheters in Stage III-V CKD Patients	2692.5	74.4	2546.7	2838.3	5482.4	1937.4	1685.1	9279.8	5501.1	122.2	5261.6	5740.6	5379.4	121.6	5141.0	5617.7	5797.8	151.8	5500.2	6095.4
SNP04-NSAIDs for Hypertension, Heart failure or Chronic Kidney Disease	4652.3	29.9	4593.6	4710.9	3058.7	26.1	3007.5	3109.9	2216.5	18.4	2180.4	2252.5	1922.1	17.9	1887.1	1957.2	1703.8	0.3	1703.2	1704.4
Total	26928.3	345.9	26250.3	27606.2	30471.3	2003.7	26544.1	34398.4	27178.6	494.0	26210.4	28146.9	26937.4	537.4	25884.2	27990.7	27490.6	196.4	27105.7	27875.5

© 2021 Mafi JN et al. *JAMA Network Open.*

Line-level spending trends per 1,000 Medicare Beneficiaries among 10 measures with no ICD9 or ICD10 codes used to classify "not wasteful" services																									
Measure	2014 Line Spend	std_err_linespend_2014	CI 2014		2015 Line Spend	std_err_linespend_2015	CI 2015		2016 Line Spend	std_err_linespend_2016	CI 2016		2017 Line Spend	std_err_linespend_2017	CI 2017		2018 Line Spend	std_err_linespend_2018	CI 2018	
AI03-Diagnostic Testing for Chronic Urticaria	7.9	0.5	7.0	8.9	7.2	0.4	6.4	8.1	10.4	0.6	9.3	11.6	8.5	0.4	7.7	9.4	7.2	3.0	1.2	13.1
APA01-Concurrent Use of Two or More Antipsychotic Medications	7816.6	328.5	7172.7	8460.5	7251.8	339.0	6587.4	7916.2	5975.4	315.0	5358.0	6592.7	6338.2	340.4	5671.0	7005.5	6555.4	13.1	6529.7	6581.0
ASA02-Preoperative Cardiac Echocardiography or Stress Testing	79.5	1.7	76.2	82.8	67.6	1.4	64.8	70.3	65.0	1.2	62.7	67.3	64.9	1.2	62.6	67.2	62.6	9.3	44.3	80.8
DOR21-Arthroscopic Lavage and Debridement for Knee Osteoarthritis	149.6	3.4	143.0	156.2	152.4	3.1	146.3	158.6	146.2	3.3	139.7	152.7	126.8	2.8	121.3	132.4	124.7	33.6	58.8	190.6
DOR85-Antidepressants Monotherapy in Bipolar Disorder	58.0	2.8	52.4	63.5	52.2	4.3	43.8	60.6	50.5	4.0	42.5	58.4	52.5	5.2	42.2	62.8	50.0	3.3	43.4	56.6
GE01-Colorectal Cancer Screening in Adults 50 Years and Older	6085.0	19.8	6046.2	6123.8	5968.7	18.9	5931.6	6005.7	5748.5	17.7	5713.9	5783.1	5347.1	17.2	5313.5	5380.8	5524.8	0.8	5523.3	5526.4
SCCT01-Coronary Artery Calcium Scoring for Known CAD	33.3	2.4	28.5	38.0	36.2	2.2	31.9	40.5	36.3	2.0	32.3	40.3	51.3	2.1	47.2	55.4	60.8	11.6	38.0	83.6
SCP05-Bleeding Time Testing	1.6	0.1	1.4	1.7	1.1	0.1	1.0	1.3	0.7	0.0	0.7	0.8	0.6	0.0	0.5	0.6	0.4	0.3	-0.3	1.0
SNP01-Peripherally Inserted Central Catheters in Stage III-V CKD Patients	1610.2	69.0	1475.0	1745.4	2252.4	117.1	2022.9	2481.9	3940.8	111.3	3722.6	4159.1	3626.2	106.9	3416.7	3835.7	3596.0	122.4	3356.2	3835.9
SNP04-NSAIDs for Hypertension, Heart failure or Chronic Kidney Disease	4652.2	29.9	4593.6	4710.8	3058.7	26.1	3007.5	3109.9	2216.5	18.4	2180.4	2252.5	1922.2	17.9	1887.1	1957.2	1703.8	0.3	1703.3	1704.4
Total	20493.8	337.6	19832.0	21155.6	18848.2	360.1	18142.4	19554.1	18190.4	335.1	17533.6	18847.1	17538.4	357.7	16837.2	18239.5	17685.7	128.5	17433.8	17937.6

© 2021 Mafi JN et al. *JAMA Network Open.*

Line-level spending trends per 1,000 Medicare Beneficiaries among 8 measures with no ICD9 or ICD10 codes used to classify "not wasteful" services																				
Measure	2014 Services	std_err_services_2014	CI 2014		2015 Services	std_err_services_2015	CI 2015		2016 Services	std_err_services_2016	CI 2016		2017 Services	std_err_services_2017	CI 2017		2018 Services	std_err_services_2018	CI 2018	
AI03-Diagnostic Testing for Chronic Urticaria	0.09	0.00	0.09	0.09	0.09	0.00	0.09	0.09	0.10	0.00	0.10	0.10	0.11	0.00	0.11	0.11	0.11	0.00	0.11	0.11
APA01-Concurrent Use of Two or More Antipsychotic Medications	32.56	0.70	31.19	33.93	31.09	0.66	29.79	32.39	29.55	0.63	28.32	30.78	29.31	0.63	28.08	30.54	28.15	0.02	28.11	28.19
DOR21-Arthroscopic Lavage and Debridement for Knee Osteoarthritis	0.11	0.00	0.11	0.11	0.11	0.00	0.11	0.11	0.11	0.00	0.11	0.11	0.10	0.00	0.10	0.10	0.09	0.00	0.08	0.10
DOR85-Antidepressants Monotherapy in Bipolar Disorder	0.90	0.01	0.87	0.93	0.96	0.02	0.93	0.99	0.89	0.02	0.86	0.92	0.93	0.02	0.89	0.97	0.95	0.02	0.92	0.98
SCCT01-Coronary Artery Calcium Scoring for Known CAD	0.09	0.00	0.09	0.09	0.12	0.00	0.12	0.12	0.15	0.00	0.15	0.15	0.20	0.00	0.20	0.20	0.22	0.00	0.22	0.22
SCP05-Bleeding Time Testing	0.26	0.00	0.25	0.27	0.21	0.01	0.20	0.22	0.12	0.00	0.11	0.13	0.09	0.00	0.09	0.09	0.07	0.03	0.02	0.12
SNP01-Peripherally Inserted Central Catheters in Stage III-V CKD Patients	0.74	0.00	0.73	0.75	0.79	0.00	0.78	0.80	0.86	0.00	0.85	0.87	0.89	0.00	0.88	0.90	0.93	0.00	0.92	0.94
SNP04-NSAIDs for Hypertension, Heart failure or Chronic Kidney Disease	58.39	0.09	58.21	58.57	60.77	0.09	60.60	60.94	61.52	0.08	61.35	61.69	60.32	0.08	60.17	60.47	56.94	0.00	56.94	56.94
Total	93.14	0.70	91.76	94.52	94.14	0.67	92.83	95.45	93.30	0.63	92.06	94.54	91.95	0.63	90.71	93.19	87.46	0.04	87.39	87.53

© 2021 Mafi JN et al. *JAMA Network Open.*

Claim-level spending trends per 1,000 Medicare Beneficiaries among 8 measures with no ICD9 or ICD10 codes used to classify "not wasteful" services

Measure	2014 Claim Spend	std_err_claimspend_2014	CI 2014		2015 Claim Spend	std_err_claimspend_2015	CI 2015		2016 Claim Spend	std_err_claimspend_2016	CI 2016		2017 Claim Spend	std_err_claimspend_2017	CI 2017		2018 Claim Spend	std_err_claimspend_2018	CI 2018	
AI03-Diagnostic Testing for Chronic Urticaria	29.9	3.3	23.5	36.4	36.1	5.6	25.1	47.0	35.8	3.7	28.4	43.1	47.8	4.3	39.4	56.3	44.4	43.6	-41.1	129.9
APA01-Concurrent Use of Two or More Antipsychotic Medications	7816.9	328.5	7173.0	8460.8	10671.3	507.7	9676.1	11666.4	8971.6	476.9	8036.9	9906.3	9548.7	521.4	8526.7	10570.7	9912.0	20.3	9872.1	9951.9
DOR21-Arthroscopic Lavage and Debridement for Knee Osteoarthritis	149.6	3.4	143.0	156.2	152.4	3.1	146.3	158.6	146.2	3.3	139.7	152.7	126.8	2.8	121.3	132.4	124.7	33.6	58.8	190.6
DOR85-Antidepressants Monotherapy in Bipolar Disorder	58.0	2.8	52.4	63.5	52.2	4.3	43.8	60.6	50.5	4.0	42.5	58.4	52.5	5.2	42.2	62.8	50.0	3.3	43.4	56.6
SCCT01-Coronary Artery Calcium Scoring for Known CAD	38.9	2.5	34.1	43.7	43.1	2.2	38.7	47.4	44.3	2.1	40.3	48.4	62.1	2.1	57.9	66.2	72.7	11.8	49.6	95.7
SCP05-Bleeding Time Testing	318.9	17.3	285.0	352.8	251.1	16.3	219.1	283.0	124.1	8.5	107.4	140.8	121.3	11.1	99.6	143.1	107.2	106.6	-101.9	316.2
SNP01-Peripherally Inserted Central Catheters in Stage III-V CKD Patients	2692.5	74.4	2546.7	2838.3	5482.4	1937.4	1685.1	9279.8	5501.1	122.2	5261.6	5740.6	5379.4	121.6	5141.0	5617.7	5797.8	151.8	5500.2	6095.4
SNP04-NSAIDs for Hypertension, Heart failure or Chronic Kidney Disease	4652.3	29.9	4593.6	4710.9	3058.7	26.1	3007.5	3109.9	2216.5	18.4	2180.4	2252.5	1922.1	17.9	1887.1	1957.2	1703.8	0.3	1703.2	1704.4
Total	15757.0	338.7	15093.2	16420.7	19747.2	2003.1	15821.1	23673.3	17090.0	492.8	16124.2	18055.8	17260.7	535.9	16210.4	18311.1	17812.6	195.0	17430.4	18194.7

© 2021 Mafi JN et al. *JAMA Network Open.*

Line-level spending trends per 1,000 Medicare Beneficiaries among 8 measures with no ICD9 or ICD10 codes used to classify "not wasteful" services

Measure	2014 Line Spend	std_err_linespend_2014	CI 2014		2015 Line Spend	std_err_linespend_2015	CI 2015		2016 Line Spend	std_err_linespend_2016	CI 2016		2017 Line Spend	std_err_linespend_2017	CI 2017		2018 Line Spend	std_err_linespend_2018	CI 2018	
AI03-Diagnostic Testing for Chronic Urticaria	7.9	0.5	7.0	8.9	7.2	0.4	6.4	8.1	10.4	0.6	9.3	11.6	8.5	0.4	7.7	9.4	7.2	3.0	1.2	13.1
APA01-Concurrent Use of Two or More Antipsychotic Medications	7816.6	328.5	7172.7	8460.5	7251.8	339.0	6587.4	7916.2	5975.4	315.0	5358.0	6592.7	6338.2	340.4	5671.0	7005.5	6555.4	13.1	6529.7	6581.0
DOR21-Arthroscopic Lavage and Debridement for Knee Osteoarthritis	149.6	3.4	143.0	156.2	152.4	3.1	146.3	158.6	146.2	3.3	139.7	152.7	126.8	2.8	121.3	132.4	124.7	33.6	58.8	190.6
DOR85-Antidepressants Monotherapy in Bipolar Disorder	58.0	2.8	52.4	63.5	52.2	4.3	43.8	60.6	50.5	4.0	42.5	58.4	52.5	5.2	42.2	62.8	50.0	3.3	43.4	56.6
SCCT01-Coronary Artery Calcium Scoring for Known CAD	33.3	2.4	28.5	38.0	36.2	2.2	31.9	40.5	36.3	2.0	32.3	40.3	51.3	2.1	47.2	55.4	60.8	11.6	38.0	83.6
SCP05-Bleeding Time Testing	1.6	0.1	1.4	1.7	1.1	0.1	1.0	1.3	0.7	0.0	0.7	0.8	0.6	0.0	0.5	0.6	0.4	0.3	-0.3	1.0
SNP01-Peripherally Inserted Central Catheters in Stage III-V CKD Patients	1610.2	69.0	1475.0	1745.4	2252.4	117.1	2022.9	2481.9	3940.8	111.3	3722.6	4159.1	3626.2	106.9	3416.7	3835.7	3596.0	122.4	3356.2	3835.9
SNP04-NSAIDs for Hypertension, Heart failure or Chronic Kidney Disease	4652.2	29.9	4593.6	4710.8	3058.7	26.1	3007.5	3109.9	2216.5	18.4	2180.4	2252.5	1922.2	17.9	1887.1	1957.2	1703.8	0.3	1703.3	1704.4
Total	14329.3	337.1	13668.7	14989.9	12812.0	359.6	12107.2	13516.9	12376.8	334.6	11721.0	13032.7	12126.3	357.3	11426.0	12826.6	12098.3	128.2	11847.0	12349.6

© 2021 Mafi JN et al. *JAMA Network Open.*

REFERENCES

1. VanderWeele TJ, Ding P. Sensitivity Analysis in Observational Research: Introducing the E-Value. *Ann Intern Med.* 2017;167(4):268-274.

© 2021 Mafi JN et al. *JAMA Network Open.*
